# The Role of Dendritic Spines in Water Exchange Measurements With Diffusion MRI: Time‐Dependent Single Diffusion Encoding MRI

**DOI:** 10.1002/nbm.70382

**Published:** 2026-09-11

**Authors:** Kadir Şimşek, Arthur Chakwizira, Markus Nilsson, Marco Palombo

**Affiliations:** ^1^ Cardiff University Brain Research Imaging Centre (CUBRIC) Cardiff University Cardiff UK; ^2^ School of Computer Science and Informatics Cardiff University Cardiff UK; ^3^ Medical Radiation Physics, Lund Lund University Lund Sweden; ^4^ Department of Clinical Sciences Lund, Radiology Lund University Lund Sweden

**Keywords:** dendritic spines, diffusion, exchange, microstructure, modelling, MRI, time‐dependence

## Abstract

Time‐dependent diffusion MRI (dMRI) using single diffusion encoding (SDE) is sensitive to water dynamics in biological tissues, yet interpreting its signals requires careful consideration of underlying microstructure. While prior work has focused on restricted/hindered diffusion and membrane permeation, additional exchange mechanisms such as diffusion‐mediated exchange between dendritic shaft and spines in gray matter (GM) remain understudied. Here, we hypothesize that water diffusion within impermeable spiny dendrites can produce time‐dependent SDE signals indistinguishable from those arising from permeative exchange; and assess to what extent spine density impacts estimates of exchange time. Using Monte Carlo simulations and analytical solutions from the narrow escape problem, we quantify spine–shaft and shaft–spine exchange times, revealing characteristic times (1–50 ms) comparable to permeative exchange estimates in the cortex. We show that a modified two‐compartment Kärger model accurately captures the time‐dependent SDE signal along spiny dendrites but yields exchange estimates that reflect total spine volume fraction rather than specific spine morphology. Simulations reveal that unaccounted diffusion‐mediated exchange from dendritic spines substantially biases Neurite Exchange Imaging (NEXI) estimates, inflating the apparent exchange rate and the inferred membrane permeability in proportion to spine volume fraction. Further, we propose an extended three‐compartment Kärger model (and its coarse‐grained version) incorporating both diffusion‐mediated exchange between dendritic shaft and spines and permeative exchange with extracellular space. Critically, while the three‐compartment Kärger model and its coarse‐grained version capture both exchange mechanisms, they cannot uniquely disentangle membrane permeability from spine volume fraction. Finally, we highlight the importance of accounting for dendritic spines when inferring membrane permeability from diffusion MRI exchange rates, as the additional membrane area contributed by spines increases the effective dendritic surface‐to‐volume ratio by approximately 24%–60% for realistic spine densities and, if neglected, leads to a corresponding overestimation of the true membrane permeability. These findings underscore the necessity of considering dendritic spine contributions when interpreting time‐dependent SDE data and caution against attributing exchange effects solely to membrane permeability. Our study further highlights the need for advanced acquisition and modeling approaches to differentiate permeative and diffusion‐mediated geometric exchange in GM.

## Graphical abstract

Geometric exchange between dendritic spines and shafts is often neglected, yet it exhibits the same signature as permeative exchange, biasing NEXI/SMEX measurements. We propose two models: an extended three‐compartment Kärger model that resolves spine–shaft exchange explicitly, and a coarse‐grained Kärger model that absorbs spines into an effective cylinder radius.

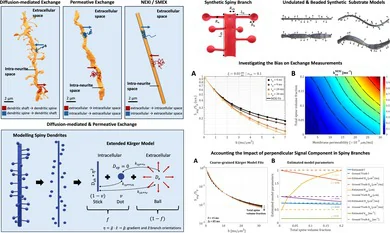

**Abbreviations**
δ
: diffusion gradient duration
Δ
: diffusion gradient separation time
$\Delta k_{\mathrm{sp}\to \mathrm{sh}}$
: percentage change of spine‐to‐shaft exit rate
ε: dimensionless modified pore size
N
: concentration curve of spins
θ
: angle between diffusion gradient and dendritic shaft
$\lambda_{\mathrm{und}}$
: undulation wavelength
ν
: spine volume fraction
$\nu_{\mathrm{tot}}$
: total spine volume fraction
ξ
: membrane permeability
ϑ
: power‐law exponent
ρ
: spine density along the dendritic shaft
σ: pore area density
χ^2^
: goodness of model fitting
ζ
: measurable sensitivity parameter
$A_{\text{bead}}$
: beading amplitude
AIC
: Akaike information criterion
CA1: hippocampal cornu ammonis 1
$D_{0}$
: free bulk diffusivity
$D_{e}$
: extracellular diffusivity
$D_{i}$
: intracellular diffusivity
$D_{\text{in}}$
: intra‐neurite diffusivity
$D_{\mathrm{sh}}$
: diffusivity in dendritic shaft
$D_{\mathrm{sp}}$
: diffusivity in dendritic spines
DDE: double diffusion encoding
DEXSY: diffusion exchange spectroscopy
DM: diffusion‐mediated exchange
dMRI: diffusion‐weighted MR imaging
EM: electron microscopy
f
: intracellular volume fraction
FWF: free gradient waveform
$\vec{g}$
: diffusion gradient vector
GM: gray matter
k
: exchange rate
K
: kurtosis
$K_{\infty }$
: kurtosis at very long diffusion times
$k_{\mathrm{ex}}^{\mathrm{DM}}$
: diffusion‐mediated exchange rate
$k_{e\to i}$
: exchange rate from extra‐to‐intracellular space
$k_{i\to e}$
: exchange rate from intra‐to‐extracellular space
$k_{\mathrm{sh}\to \mathrm{sp}}$
: shaft‐to‐spine exchange rate
$k_{\mathrm{sp}\to \mathrm{sh}}$
: spine‐to‐shaft exchange rate
$l_{c}$
: correlation length
$L_{n}$
: spine neck length
$L_{\mathrm{sh}}$
: dendritic shaft length
$\vec{n}$
: neurite orientation
$N_{s}$
: number of spins
$N_{t}$
: number of time steps
$N_{\mathrm{und}}$
: undulation period along the shaft
NEXI: Neurite Exchange Imaging
np: narrow pulse
PGSE: pulsed gradient spin echo
$R_{\mathrm{eff}}$
: effective radius
$R_{h}$
: spine head radius
$R_{n}$
: spine neck radius
$R_{\mathrm{sh}}$
: dendritic shaft radius
$S_{\mathrm{avg}}$
: powder‐averaged signal
$S_{\|}$
: diffusion signal parallel to the dendritic shaft
$S_{⊥}$
: diffusion signal perpendicular to the dendritic shaft
$S_{e}$
: diffusion signal in the extracellular space
$S_{\mathrm{sh}}$
: diffusion signal in the dendritic shaft
$S_{\mathrm{sp}}$
: diffusion signal in the dendritic spine
SDE: single diffusion encoding
SMEX: Standard Model with Exchange
SV
: surface‐to‐volume ratio
$t_{\mathrm{ex}}^{\text{NEXI}}$
: estimated exchange time with NEXI
$t_{d}$
: diffusion time
$t_{\mathrm{ex}}$
: exchange time
$t_{\mathrm{ex}}^{\mathrm{DM}}$
: diffusion‐mediated exchange time
WM: white matter
wp: wide pulse

## 1 Introduction

Time‐dependent diffusion MRI (dMRI) is a powerful technique that probes the temporal dynamics of molecular diffusion within biological tissues, offering insights into both restriction and exchange [1, 2, 3, 4, 5, 6, 7, 8, 9, 10, 11, 12, 13, 14, 15, 16, 17, 18, 19, 20, 21, 22]. Numerous methods have been proposed to quantify exchange rates of water molecules in the brain [19, 20, 23, 24, 25, 26, 27, 28, 29, 30, 31, 32, 33, 34, 35, 36, 37]. Some studies investigated exchange rates using model‐based approaches, under the assumption that permeative exchange across the cellular membrane is the most relevant exchange process. While permeative exchange in white matter (WM) is expected to be slow and negligible due to the insulating myelin sheath surrounding axons [14, 38, 39, 40], it is likely faster and nonnegligible in gray matter (GM), where myelinated neurites (axons and dendrites) are far less abundant. Two recent examples of such model‐based methods, designed to characterize permeative exchange in GM, are the Neurite Exchange Imaging (NEXI) [14] and the Standard Model with Exchange (SMEX) [13]. Both NEXI and SMEX rely on the pulsed gradient spin echo (PGSE) acquisitions [41] with multiple diffusion times, that is, single diffusion encoding (SDE) measurements with different diffusion times. Using NEXI/SMEX, recent works characterized water exchange in GM, reporting exchange times ranging from a few milliseconds to hundreds of milliseconds [13, 14, 42, 43], which are comparable to the typical diffusion times used in SDE measurements.

However, the SDE signal at varying diffusion times carries entangled information on both restriction and exchange processes [2, 8, 13, 14, 19, 20, 42, 43]; therefore, disentangling and estimating diffusion metrics specific to either restriction or exchange remains degenerate [6, 16, 32, 44, 45]. Recent studies exploited advanced diffusion encoding techniques to address this degeneracy in time‐dependent SDE measurements. One approach involves double diffusion encoding (DDE) measurements, which help to disentangle this degeneracy by introducing additional experimental dimensions [2, 25, 35, 46, 47]. Other studies use diffusion exchange spectroscopy (DEXSY) methods to isolate distinct water pools with different restriction and exchange characteristics and quantify the corresponding characteristic restriction size and exchange times [34, 35, 37]. Others modulated the DDE blocks to isolate different exchange rates and characterize them [24, 25, 29]. In addition to DDE methods, more complex encoding schemes like free gradient waveforms (FWF) have been utilized to disentangle restriction and exchange in the brain, particularly in the GM [31, 32, 48]. One recent study mapped how sensitive the diffusion signal acquired with FWF encoding is to restriction and exchange effects and provided sequence optimization for determining the interplay between restriction and exchange effects [31].

While advanced diffusion encodings (beyond SDE) show promise for disentangling restriction and exchange, time‐dependent SDE measurements remain clinically appealing due to their experimental robustness [49]. However, interpreting these measurements requires careful consideration of the underlying microstructure. Although time‐dependent SDE signals decreasing with time at a fixed *b* value are often attributed to permeative exchange, growing evidence suggests contributions from other exchange mechanisms. Some recent studies reported on potential alternative exchange mechanisms beside permeability, for example, geometric exchange within impermeable compartments, and vascular water exchange [13, 23, 24, 30, 31, 35, 50, 51] and some preliminary works suggest that dendritic spines (small, protruding structures on the dendrites of neurons), due to their restrictive geometry, may exhibit time‐dependent signatures resembling permeative exchange [52, 53, 54], highlighting the need to distinguish these mechanisms in SDE‐based analyses.

The aim of this work is to investigate the degree to which subcellular structures such as dendritic spines contribute to estimates of exchange in GM from time‐dependent SDE measurements. In another connected and complementary work [55], we aim instead to investigate the same mechanisms beyond SDE encodings.

**FIGURE 1:**
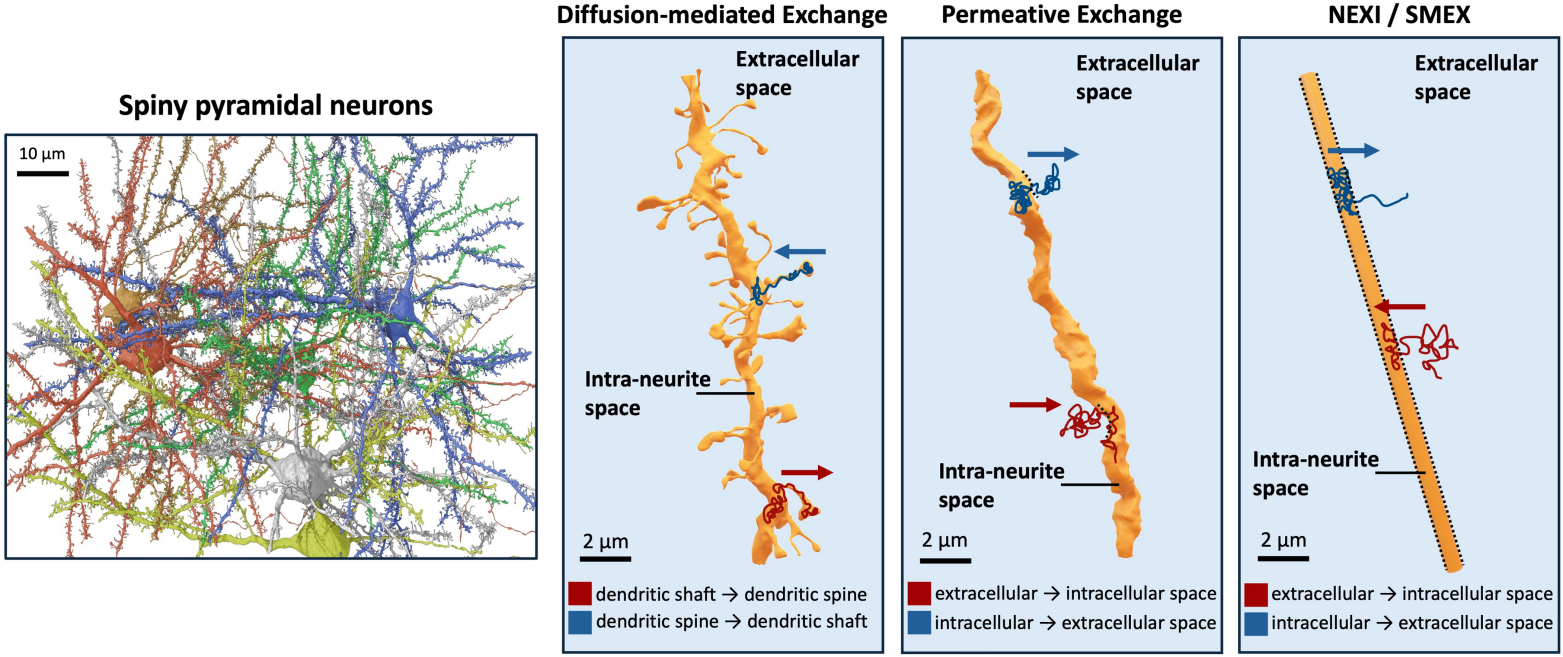
Illustration of spiny dendritic branches and different diffusion exchange mechanisms. The diffusion exchange mechanism is often considered as permeative exchange across cell membrane between intracellular and extracellular compartments. The NEXI and SMEX are the well‐known biophysical models to estimate exchange times between these compartments. Here we introduce another mechanism contributing to the exchange estimates, referred to as diffusion‐mediated exchange which is the exchange between intracellular subcompartments (e.g., between dendritic spines and dendritic shaft).

Here, we hypothesize that water diffusing within spiny dendrites in the GM, without crossing the cell membrane, can impart a time‐dependent signature on SDE measurements which is similar to or possibly indistinguishable from permeative exchange (see Figure 1). Moreover, we propose an extended Kärger model of three compartments: dendritic shaft, spine and extracellular space, inspired by a previous model for nerves [56]. The signal from spins diffusing within the shaft is modeled as diffusion within sticks. The signal within the spines is treated as fully restricted, representing still water, while the extracellular space is modeled as isotropic Gaussian diffusion. All compartments interact and exchange with each other. Using Monte Carlo simulations and digital substrates of spiny neurites, we test our hypothesis, evaluate the impact of different spine densities and morphologies on the diffusion exchange mechanisms and compare it with permeative exchange estimates using NEXI and SMEX.

## 2 Theory

In this section, our objective is to develop a theoretical framework characterizing both permeative and diffusion‐mediated exchange processes. By deriving analytical predictions, we will establish a benchmark for interpreting our results from Monte Carlo simulations (described in the Methods section). This approach will enable us to elucidate the interplay between these two exchange mechanisms in time‐dependent SDE measurements, providing deeper insights into their relative contributions and dynamics.

We first provide a brief overview of the dendritic spines' morphology from existing literature and then introduce theoretical approaches to characterize both molecular diffusion in smooth dendrites subject to permeative exchange across the dendritic membrane, and molecular diffusion in spiny dendrites, accounting for diffusion‐mediated exchange between dendritic shaft and spines.

### 2.1 Dendritic Spines: Morphological Diversity and Regional Variations

Dendritic spines are small, protruding structures on the dendrites of neurons which were first revealed by Cajal using Golgi staining technique [57]. They exhibit diverse morphologies, including thin, stubby, and mushroom‐shaped forms [58, 59, 60], which are associated with synaptic strength and plasticity [61, 62, 63, 64]. Their shape and density can vary in response to neural activity, influencing cognitive functions and neurological disorders across the whole brain [63, 65, 66, 67]. On average, a dendritic spine has a volume ranging from 0.01 to 0.8 ${\mathrm{\mu m}}^{3}$, with surface area varying between 0.1 and $5\;\mu m^{2}$ [65, 68, 69]. The spine head radius, often associated with synaptic strength, typically ranges from 0.15 to 0.55μm, with mushroom spines having the largest spine heads [68, 70, 71]. The spine neck has a length ranging from 0.1 to 2μm and a radius ranging from 0.02 to 0.25μm [60, 69, 70]. The dendritic spine sizes offer a structural basis for understanding synaptic integration and plasticity.

The spine density on the neurons ranges between 1 and 10 spines per micron ($\mu m^{‐1}$) [65, 72, 73, 74]. For instance, hippocampal thick apical dendrites of CA1 pyramidal neurons near the stratum radiatum can exhibit spine density up to $10\;\mu m^{‐1}$ [65, 75, 76, 77, 78] while the pyramidal neurons in the visual cortex have spine densities around $1\;\mu m^{‐1}$ or less [79, 80]. The pyramidal neurons in the cortical layers can exhibit different spine densities due to their distinct dynamics [81, 82, 83, 84]. The spiniest type of neurons is the cerebellar Purkinje cells. 3D electron‐microscopy (EM) analysis revealed that the spine density can reach up to $15\;\mu m^{‐1}$ in the dendritic branches of Purkinje cells [65, 85]. In the brain cortex, the estimated synapse density is approximately $7.6\times 10^{8}$ synapses per ${\mathrm{mm}}^{3}$ [86, 87]. Assuming each synapse is associated with a dendritic spine of mean volume $0.1\;\mu m^{3}$ [60, 88, 89, 90], we would expect a total volume fraction occupied by spines (ν) in the brain cerebral cortex of $7.6\times 10^{8}*0.1\times 10^{-9}∼8\%$. This aligns with reported total volume fractions of dendritic spines in GM of brain cortex over different species [91]. A similar calculation could be done for the cerebellar cortex where the spiniest Purkinje cells form complex synaptic wiring; however, the literature reporting on the synaptic density in the cerebellar cortex is discordant and here we can only suggest that the total volume fraction occupied by spines in cerebellar cortex should be higher than ∼8%.

### 2.2 The Narrow Escape Problem and Diffusion‐Mediated Exchange in Dendritic Spines

The molecular diffusion in membrane‐enclosed cellular domains with narrow openings has been extensively investigated in the literature. The mathematical problem involving diffusing particles escaping through a narrow channel is referred to as the “Narrow Escape Problem” [92, 93, 94, 95, 96, 97, 98]. This problem quantifies the escape rate of a particle from a composite domain containing a small circular absorbing surface. Several studies have applied this framework to describe the mean diffusive exit rate of particles within dendritic spines and shafts [92, 99, 100, 101, 102, 103, 104]. These works have primarily focused on theoretical development and Monte Carlo simulations of one directional diffusive exchange rates from the dendritic spines or shafts. In this study, we leverage the derived analytical solutions to characterize diffusion‐mediated exchange between dendritic spines and shaft.

Considering a dendritic spine having a spherical head with radius $R_{h}$, connected to the shaft via cylindrical neck with a length $L_{n}$ and radius $R_{n}$, the mean diffusive exit rate of particles with bulk diffusivity $D_{0}$ from the dendritic spine to the shaft is given by [101, 102]

(1) $$k_{\mathrm{sp}\to \mathrm{sh}}=\frac{6D_{0}R_{n}^{2}}{L_{n}\cdot \left(2\pi R_{h}^{3}+3R_{n}^{2}L_{n}+2\pi R_{h}^{2}R_{n}\ln\left(\frac{R_{h}}{R_{n}}\right)-2R_{n}^{3}\right)\;}$$

The diffusive exit rate of a particle inside dendritic spines, as described in Equation (1), represents only one component of the overall diffusion‐mediated exchange. To complete this framework, it is necessary to characterize the complementary process: diffusion from the dendritic shaft into the spine. This process is equally important, as the diffusion exchange measured in dMRI is inherently bidirectional between compartments and therefore reflects the combined contributions of both spine‐to‐shaft and shaft‐to‐spine exchange.

This problem can be considered as an aspect of the narrow escape problem and has been studied analytically for various geometric domains [92, 93, 95, 105]. One recent work of relevance for this study solved the problem for cylindrical domains [103]. The proposed solution (eq. 7 in [103]) depends on the pore area density (σ) and the asymptotic ratio between $R_{n}$ and the dendritic shaft radius $R_{\mathrm{sh}}$, $\varepsilon =\frac{\pi R_{n}}{4R_{\mathrm{sh}}}$ (valid for $R_{n}\ll R_{\mathrm{sh}}$), to satisfy the approximation in Berg and Purcell's original model for the narrow escape problem [95]. Using this framework, the analytical expression for the diffusive exit rate from shaft‐to‐spine, $k_{\mathrm{sh}\to \mathrm{sp}}$, is given by (eq.7 in [103]):

(2) $$k_{\mathrm{sh}\to \mathrm{sp}}=\frac{8D_{0}}{R_{\mathrm{sh}}^{2}}\frac{2\sigma }{2\sigma +4\varepsilon \left(1-\sigma \right)\left(2-\varepsilon \right)}$$

The pore area density σ can be easily computed from the ratio between the total spine neck area on the dendritic shaft and the surface area of dendritic shaft, yielding the following relation $\sigma =\frac{{\mathrm{\rho R}}_{n}^{2}}{2R_{\mathrm{sh}}}$ where ρ is the dendritic spine density (i.e., number of spines per unit length of dendritic segment) on the dendrite's branch. The Equation (2) completes the whole picture in determining the diffusion‐mediated exchange times between intracellular subcompartments.

The diffusion‐mediated exchange time between intracellular subcompartments can be computed using two analytical solutions in Equations (1) and (2). The total exchange rate of the system, $k_{\mathrm{ex}}^{\mathrm{DM}}$, will be defined by the sum of the exchange rates of each process, $k_{\mathrm{ex}}^{\mathrm{DM}}=k_{\mathrm{sp}\to \mathrm{sh}}+k_{\mathrm{sh}\to \mathrm{sp}}$. Then, a characteristic time for the diffusion‐mediated exchange of the system, $t_{\mathrm{ex}}^{\mathrm{DM}}$, can be computed as $t_{\mathrm{ex}}^{\mathrm{DM}}=\frac{1}{k_{\mathrm{ex}}^{\mathrm{DM}}}$. We used $k_{\mathrm{ex}}^{\mathrm{DM}}$ to compare it with the characteristic exchange rate for the permeative exchange of the system as defined by the Kärger model and assess to what extent diffusion‐mediated exchange can bias estimates of permeative exchange (see Methods section for more details).

### 2.3 The Kärger Model to Characterize Permeative Exchange: NEXI/SMEX

The quantification of exchange rates between different water compartments in the brain is essential for biophysical modeling of the diffusion MRI signal. When water compartments are assumed to be separated by permeable membranes, the exchange between these compartments is referred to as permeative exchange. Kärger proposed a model accounting for the exchange between two water pools [1, 106]. The model comprises two Gaussian diffusion pools with volume fractions $f_{1}$ and $f_{2}$, and includes intercompartmental exchange rates $k_{1}$ and $k_{2}$. The exchange rates satisfy the condition $f_{1}k_{1}=f_{2}k_{2}$, ensuring equilibrium of flux between compartments. Typically, for application to time‐dependent SDE measurements in biological tissues, the two compartments are assumed to be the intra and extracellular compartments, from now on defined by the subscripts *i* and *e*, respectively. The Kärger model assumes that the exchange during the diffusion encoding is negligible, meaning that the exchange time ($t_{\mathrm{ex}}=\frac{1}{k_{i\to e}+k_{e\to i}}$) is significantly longer than the duration of the diffusion encoding gradient (δ). Another assumption is that the intracellular compartment is homogenous at all times meaning that the exchange is “barrier limited” (i.e., $l_{c}^{2}\ll \frac{D_{i}}{k_{i\to e}}$), where $l_{c}$ is the correlation length of the intracellular compartment, $D_{i}$ is the diffusivity within the compartment, and $k_{i\to e}$ is the forward exchange rate of the corresponding compartment [2, 12, 19]. In other words, the diffusing particles fully explore their local compartment before transitioning to another.

Biophysical models accounting for the exchange rates in GM that were proposed previously, such as NEXI and SMEX, rely on the Kärger model [13, 14]. They consist of two exchanging Gaussian compartments; (i) isotropically oriented neurite (stick) compartment and (ii) the extracellular compartment. The extracellular compartment is assumed to be isotropic due to overall negligible fractional anisotropy in GM [14], which reduces the dimensionality of the model ($D_{e}\equiv D_{e⊥}=D_{e∥}$). The diffusion signal can be computed as the solution of the coupled differential equations for each compartment:

(3) $$\frac{d}{\mathrm{dt}}\left[\begin{array}{c} S_{i}\left(t\right)\\ S_{e}\left(t\right) \end{array}\right]=\left(\left[\begin{array}{cc} -k_{i\to e} & k_{e\to i}\\ k_{i\to e} & -k_{e\to i} \end{array}\right]-q^{2}\left(t\right)\left[\begin{array}{cc} D_{i}\eta^{2} & 0\\ 0 & D_{e} \end{array}\right]\right)\left[\begin{array}{c} S_{i}\left(t\right)\\ S_{e}\left(t\right) \end{array}\right]$$

where $\eta =\hat{g}\cdot \hat{n}$ and $q\left(t\right)=\gamma \int_{0}^{t}g\left(t^{\prime }\right){\mathrm{dt}}^{\prime }$, with $g\left(t\right)=g\left(t\right)\hat{g}$ being the diffusion‐sensitizing gradient, γ the proton gyromagnetic ratio, $\hat{n}$ the main neurite's direction, and $k_{i\to e}$ and $k_{e\to i}$ representing the exchange rates respectively from intracellular to extracellular compartments and vice versa. Applying the detailed balance condition $fk_{i\to e}=\left(1-f\right)k_{e\to i}$ for the two Gaussian pools (e.g., neurite and extracellular compartments), one can solve Equation (3) to obtain the total signal $K\left(q,t,\eta ;f,D_{i},D_{e},t_{\mathrm{ex}}\right)$ for SDE acquisition with $q\left(t\right)=q$ (i.e., narrow pulse approximation), and use it to compute numerically the direction‐averaged SDE signal [14]:

(4) $$\tilde{S}\left(q,t\right)=S_{0}\int_{0}^{1}K\left(q,t,\eta ;f,D_{i},D_{e},t_{\mathrm{ex}}\right)d\left(\eta \right)$$

(4a) $$K\left(q,t,\eta ;f,D_{i},D_{e},t_{\mathrm{ex}}\right)=f^{\prime }e^{-D_{i}^{\prime }q^{2}t}+\left(1-f^{\prime }\right)e^{-D_{e}^{\prime }q^{2}t}$$

(4b) $$\begin{array}{c} D_{i,e}^{\prime }=\frac{1}{2}\left[D_{i}\eta^{2}+D_{e}+\frac{1}{q^{2}t_{\mathrm{ex}}}\mp \sqrt{\left({\left(D_{e}-D_{i}\eta^{2}+\frac{2f-1}{q^{2}t_{\mathrm{ex}}}\right)}^{2}+\frac{4f-4f^{2}}{q^{4}t_{\mathrm{ex}}^{2}}\right)}\right] \end{array}$$

(4c) $$f^{\prime }=\frac{fD_{i}\eta^{2}+\left(1-f\right)D_{e}-D_{e}^{\prime }}{D_{i}^{\prime }-D_{e}^{\prime }}$$

where $t_{d}$ is the diffusion time ($\Delta -\frac{\delta }{3}$, where Δ is the diffusion gradient separation time and δ is its gradient duration for the SDE), and q is defined from the *b* value as $q=\sqrt{\frac{b}{t}}$. The exchange rate measured by the NEXI can be computed as $k_{\mathrm{ex}}^{\text{NEXI}}=\frac{1}{t_{\mathrm{ex}}}$. We use superscript NEXI to differentiate it from the exchange rate ($k_{\mathrm{ex}}^{\mathrm{DM}}$) measured by diffusion‐mediated exchange.

### 2.4 Modified Two‐Compartments Kärger Model to Characterize Diffusion‐Mediated Exchange Between Dendritic Shaft and Spines

In this study, we aim to investigate how well the modified Kärger model can characterize the time‐dependent SDE intracellular signal decay for a system of impermeable spiny dendrites. We also aim to investigate how accurate the estimates of exchange rates are, in the absence of a better theory to characterize diffusion‐mediated exchange. Assuming radii of dendrites below the resolution limit [107], such that the signal decay perpendicular to the dendritic axis is negligible ($S_{⊥}\approx 1$), we hypothesize that the modified Kärger model introduced by [12, 108] would effectively characterize the impact of diffusion‐mediated exchange between dendritic shaft and spines on the intracellular diffusion‐weighted signal parallel to the dendritic axis, $S_{∥}$. However, we note that the Kärger model is technically incapable of modelling the exchange in spines scenario, because the transition rate between spines and shaft is very high, meaning that the system is likely not in the barrier‐limited regime. In such a scenario, the concentration of spins that have never left the compartment falls below the equilibrium concentration, leading to a transport rate that is below the equilibrium one (and depends on time). Hence, we expect some degree of discrepancy between the exchange rates from our simulation experiments and those predicted from the modified Kärger model.

Worth noting, here we are focusing our analysis on intracellular diffusion only. The proposed modified two‐compartments Kärger model is made of one Gaussian diffusion compartment (i.e., diffusion along the shaft main axis) and one compartment of still water or dot compartment (i.e., water fully restricted inside the spines, $D_{\mathrm{sp}}=0\frac{\mu m^{2}}{\mathrm{ms}}$):

(5) $$\begin{array}{c} \frac{d}{\mathrm{dt}}\left[\begin{array}{c} S_{\mathrm{sh}}\left(t\right)\\ S_{\mathrm{sp}}\left(t\right) \end{array}\right]=\left(\left[\begin{array}{cc} -k_{\mathrm{sh}\to \mathrm{sp}} & k_{\mathrm{sp}\to \mathrm{sh}}\\ k_{\mathrm{sh}\to \mathrm{sp}} & -k_{\mathrm{sp}\to \mathrm{sh}} \end{array}\right]-q^{2}\left(t\right)\left[\begin{array}{cc} D_{\mathrm{sh}}\eta^{2} & 0\\ 0 & 0 \end{array}\right]\right)\left[\begin{array}{c} S_{\mathrm{sh}}\left(t\right)\\ S_{\mathrm{sp}}\left(t\right) \end{array}\right] \end{array}$$

with the following initial conditions: $S_{\mathrm{sp}}\left(t=0\right)=\nu$ and $S_{\mathrm{sh}}\left(t=0\right)=1-\nu$. Here, b is the *b* value and ν is the volume fraction of dendritic spines on the dendritic segment. $D_{\mathrm{sh}}$ is the diffusivity in the spiny dendritic branch. After solving the differential system in the Equation (5), the total signal can be computed as

(6) $$S=S_{\mathrm{sh}}+S_{\mathrm{sp}}=P_{1}^{\prime }\exp\left(-D_{1}^{\prime }q^{2}t\right)+P_{2}^{\prime }\exp\left(-D_{2}^{\prime }q^{2}t\right)$$

where $P_{1}^{\prime },P_{2}^{\prime },D_{1}^{\prime },$ and $D_{2}^{\prime }$ are functions of q, given by

$$\begin{array}{c} D_{1,2}^{\prime }=\frac{1}{2}\left(X_{\mathrm{sh}}^{\prime }+X_{\mathrm{sp}}^{\prime }\mp \sqrt{{\left(X_{\mathrm{sp}}^{\prime }-X_{\mathrm{sh}}^{\prime }\right)}^{2}+\frac{4k_{\mathrm{sh}\to \mathrm{sp}}k_{\mathrm{sp}\to \mathrm{sh}}}{q^{4}}}\right)\\ \;X_{\mathrm{sh}}^{\prime }=D_{\mathrm{sh}}\eta^{2}+\frac{k_{\mathrm{sh}\to \mathrm{sp}}}{q^{2}};X_{\mathrm{sp}}^{\prime }=\frac{k_{\mathrm{sp}\to \mathrm{sh}}}{q^{2}}\\ \;P_{1}^{\prime }=\frac{D_{2}^{\prime }-\left(1-\nu \right)D_{\mathrm{sh}}\eta^{2}}{D_{2}^{\prime }-D_{1}^{\prime }}\\ \;P_{2}^{\prime }=\frac{\left(1-\nu \right)D_{\mathrm{sh}}\eta^{2}-D_{1}^{\prime }}{D_{2}^{\prime }-D_{1}^{\prime }} \end{array}$$

### 2.5 Extended Kärger Model to Characterize Both Diffusion‐Mediated Exchange Between Dendritic Shaft and Spines and Permeative Exchange

**FIGURE 2:**
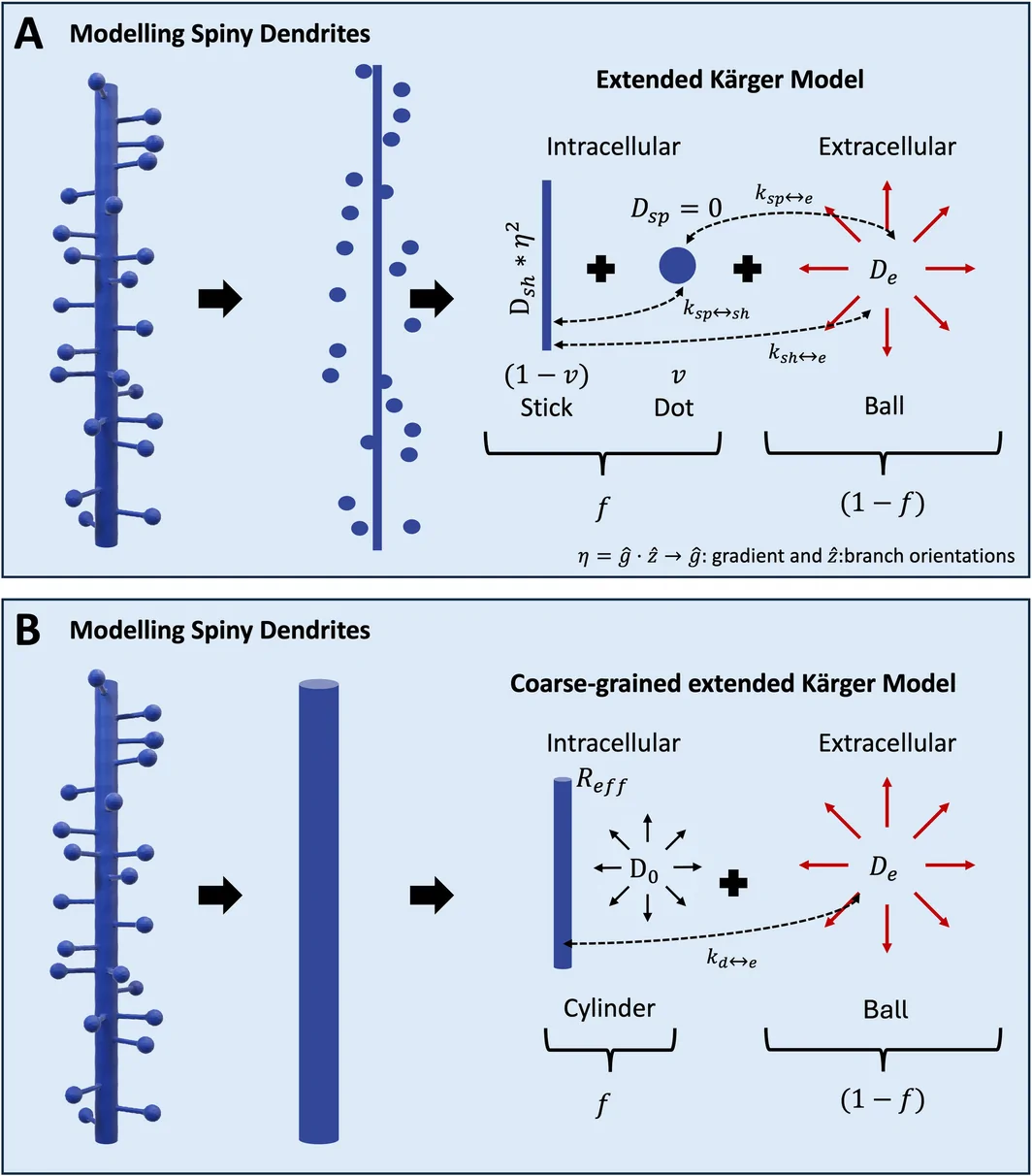
(A) Schematic of extended Kärger model. An additional compartment is introduced to accommodate dendritic spines with diffusivity $D_{\mathrm{sp}}=0$. The dendritic shaft is modelled as a stick compartment, and extracellular space is assumed to be an isotropic Gaussian compartment with diffusivity $D_{e}=1.0\frac{\mu m^{2}}{\mathrm{ms}}$. All three compartments exchange with each other at specific rates ($k_{\mathrm{sp}\to \mathrm{sh}}$: spine‐to‐shaft, $k_{\mathrm{sp}\to e}$: spine‐to‐extracellular, and $k_{\mathrm{sh}\to e}$: shaft‐to‐extracellular). (B) Schematic of coarse‐grained extended Kärger model. This model assumes that in the motional averaging regime, spiny dendritic branches become cylinders with an effective radius ($R_{\mathrm{eff}}$) with an intrinsic diffusivity ($D_{0}$) that exchange with extracellular space at a rate of $k_{d\to e}$.

A recent study conducted in vitro experiments to investigate a three‐compartment exchange system, incorporating both transmembrane exchange between intra and extracellular compartments and diffusion‐mediated exchange within the intracellular space, mimicking the exchange mechanisms in GM [109]. However, a biophysical model incorporating both the contribution of diffusion‐mediated exchange between the shaft and its dendritic spines and permeative exchange across the cell membrane is still lacking. Here, we propose an extended Kärger model as an attempt to fill this gap (see Figure 2). This model assumes the following: (i) the diffusion in the dendritic spine (spine head and neck) is fully restricted (a dot like compartment), (ii) the shaft can be modelled as stick, and (iii) extracellular diffusion can be modelled by isotropic Gaussian diffusion. All compartments exchange with each other, with rates given by their corresponding exchange rates: $k_{\mathrm{sp}\to \mathrm{sh}}$ and $k_{\mathrm{sh}\to \mathrm{sp}}$ between the dendritic spine and the shaft; $k_{\mathrm{sh}\to e}$ and $k_{e\to \mathrm{sh}}$ between dendritic shaft and the extracellular space; and $k_{\mathrm{sp}\to e}$ and $k_{e\to \mathrm{sp}}$ between the spine and the extracellular space. The extended Kärger system is then described by the following differential equation system:

(7a) $$\frac{dS_{\mathrm{sp}}}{\mathrm{dt}}=-D_{\mathrm{sp}}q^{2}S_{\mathrm{sp}}-\left(k_{\mathrm{sp}\to \mathrm{sh}}+k_{\mathrm{sp}\to e}\right)\;S_{\mathrm{sp}}+k_{e\to \mathrm{sp}}S_{e}+k_{\mathrm{sh}\to \mathrm{sp}}S_{\mathrm{sh}}$$

(7b) $$\frac{dS_{\mathrm{sh}}}{\mathrm{dt}}=-D_{\mathrm{sh}}\eta^{2}q^{2}S_{\mathrm{sh}}-\left(k_{\mathrm{sh}\to \mathrm{sp}}+k_{\mathrm{sh}\to e}\right)\;S_{\mathrm{sh}}+k_{e\to \mathrm{sh}}S_{e}+k_{\mathrm{sh}\to \mathrm{sp}}S_{\mathrm{sp}}$$

(7c) $$\frac{dS_{e}}{\mathrm{dt}}=-D_{e}q^{2}S_{e}-\left(k_{e\to \mathrm{sh}}+k_{e\to \mathrm{sp}}\right)S_{e}+k_{\mathrm{sp}\to e}S_{\mathrm{sp}}+k_{\mathrm{sh}\to e}S_{\mathrm{sh}}$$

Here, $D_{e}$, $D_{\mathrm{sh}}$, and $D_{\mathrm{sp}}$ are the corresponding diffusivities in the extracellular, dendritic shaft (with $D_{\mathrm{sh}}\eta^{2}$ yielding the axial diffusivity along the shaft where $\eta =\vec{g}\cdot \vec{n}$ the angular dependency of $D_{\mathrm{sh}}$) and dendritic spines (assuming $D_{\mathrm{sp}}=0$). $S_{e}\left(t\right),S_{\mathrm{sh}}\left(t\right)$ and $S_{\mathrm{sp}}\left(t\right)$ are the time‐dependent diffusion signals for each compartment: extracellular, dendritic shaft and dendritic spine, respectively. In Equation (7a), the signal annotation is simplified by suppressing time dependence. The corresponding exchange rates have the following relations:

(8a) $$k_{\mathrm{sp}\to e}f\nu +k_{\mathrm{sh}\to e}f\left(1-\nu \right)=\left(1-f\right)\cdot \left(k_{e\to \mathrm{sh}}+k_{e\to \mathrm{sp}}\right)$$

(8b) $$k_{\mathrm{sh}\to \mathrm{sp}}=\frac{\nu }{\left(1-\nu \right)}k_{\mathrm{sp}\to \mathrm{sh}}$$

(8c) $$k_{e\to \mathrm{sp}}=\frac{\nu f}{\left(1-f\right)}k_{\mathrm{sp}\to e}$$

(8d) $$k_{e\to \mathrm{sh}}=\frac{\left(1-\nu \right)f}{\left(1-f\right)}k_{\mathrm{sh}\to e}$$

The parameters f and ν are the volume fractions of intracellular and the dendritic spine compartments, respectively. We solved the differential system Equation (7a) numerically to obtain the directional total diffusion signal $K=S_{e}+S_{\mathrm{sp}}+S_{\mathrm{sh}}$ and then computed numerically the corresponding direction‐averaged signal as in Equation (4).

### 2.6 Coarse‐Grained Extended Kärger Model: An Adaptation to the Motional Narrowing Regime

In the coarse‐graining regime, water molecules can diffuse sufficiently fast to probe many microscopic environments during the diffusion time. Consequently, fine structural heterogeneities in tissue are averaged out and dMRI signal becomes sensitive to global microstructures in the tissue [16, 110, 111]. For instance, neurites are effectively modelled as sticks with no decay in the transverse direction ($S_{⊥}=1$) [13, 14, 16, 39, 112, 113, 114]. However, dendritic spines with total length (head + neck) up to 3μm [60, 69] introduce nonnegligible restriction effects in the perpendicular direction that can become measurable [107]. In such conditions, we can assume that the global effect of dendritic spines might turn the spiny dendritic branches into cylinders with effective radius ($R_{\mathrm{eff}}$) and effective diffusivity ($D_{0}$). This assumption enables us to reconfigure our extended Kärger model as exchange between two compartments: cylindrical dendrites with radius $R_{\mathrm{eff}}$ and intrinsic diffusivity $D_{0}$ in exchange with the extracellular space at a rate of $k_{d\to e}$ as illustrated in Figure 2B.

This reconfiguration of the extended Kärger model assumes diffusion in the motional narrowing regime, that is, when the correlation time satisfies $t_{c}\ll \delta$. Under this condition, the diffusion signal loses its dependence on Δ, allowing it to be modeled as $S_{⊥}∼{\mathrm{CR}}_{\mathrm{eff}}^{4}/D_{0}$ [115]. Consequently, the extended Kärger model can be reduced to a two‐compartment Kärger model describing exchange between effective cylinders and the extracellular space. Then, we can redefine the intracellular diffusion $D_{i}$ in Equations (4) as

(9) $$D_{i}=D_{0}\eta^{2}+\frac{CR_{\mathrm{eff}}^{4}}{D_{0}}\left(1-\eta^{2}\right)$$

where $C=\frac{7}{48}\frac{1}{\delta \left(\Delta -\frac{\delta }{3}\right)}$ is a constant determined by the diffusion timing parameters. Substituting this modified $D_{i}$ into the two‐compartment Kärger or equivalently the NEXI model in Equation (3) yields an exchange framework that accounts for dendritic spines in the coarse‐grained regime. This model reduces the number of free parameters from seven to five and provides a simplified picture for a more parsimonious model of diffusion in GM.

## 3 Methods

In this section, our objective is to design virtual experiments of time‐dependent SDE measurements using Monte Carlo simulations to investigate the impact of diffusion‐mediated exchange between dendritic shaft and spines and compare our results with theoretical predictions from the previous section.

We first describe how we built digital substrates representative of spiny dendrites and then we describe our simulation experiments and analysis.

### 3.1 Spiny Dendritic Branches

We propose a simple tunable digital model of spiny branches for understanding the diffusion‐mediated exchange mechanism within intracellular compartments (i.e., between dendritic spines and dendritic shaft). The digital model represents dendritic spines with mushroom‐like morphology, because they are the most mature and stable type [116, 117]. We model dendrites and their spines by spherical spine heads connected to a cylindrical dendritic shaft through a narrow cylindrical neck [118]. The dendritic shaft is aligned in the $\hat{z}$ direction and all spines are attached to the dendritic shaft perpendicularly. The spines are placed at nodes randomly arranged along the dendritic shaft. To avoid overlapping of multiple spines at the same node, we imposed an angular separation of 90° in the plane perpendicular to the shaft between the neck of the current spine and the neck of the next one, after the first randomized spine placement. For spine densities exceeding ~$1.2\;\mu m^{‐1}$, complete randomness was unattainable with our approach, leading to regular ordered arrangement. It is worth noting that all the spiny branches from real 3D reconstructions used here had spine density ≤ $1.2\;\mu m^{‐1}$ and that regular ordered spine arrangements along dendrites are not unrealistic—and in fact they are common—for neurons with high spine density. For example, in Purkinje cells (where spine density ≥ $2\;\mu m^{‐1}$), spines are arranged in helical patterns, positioned regularly along the dendrite with constant spacing (~0.5−0.6 μm) and angular displacement between them [119].

**FIGURE 3:**
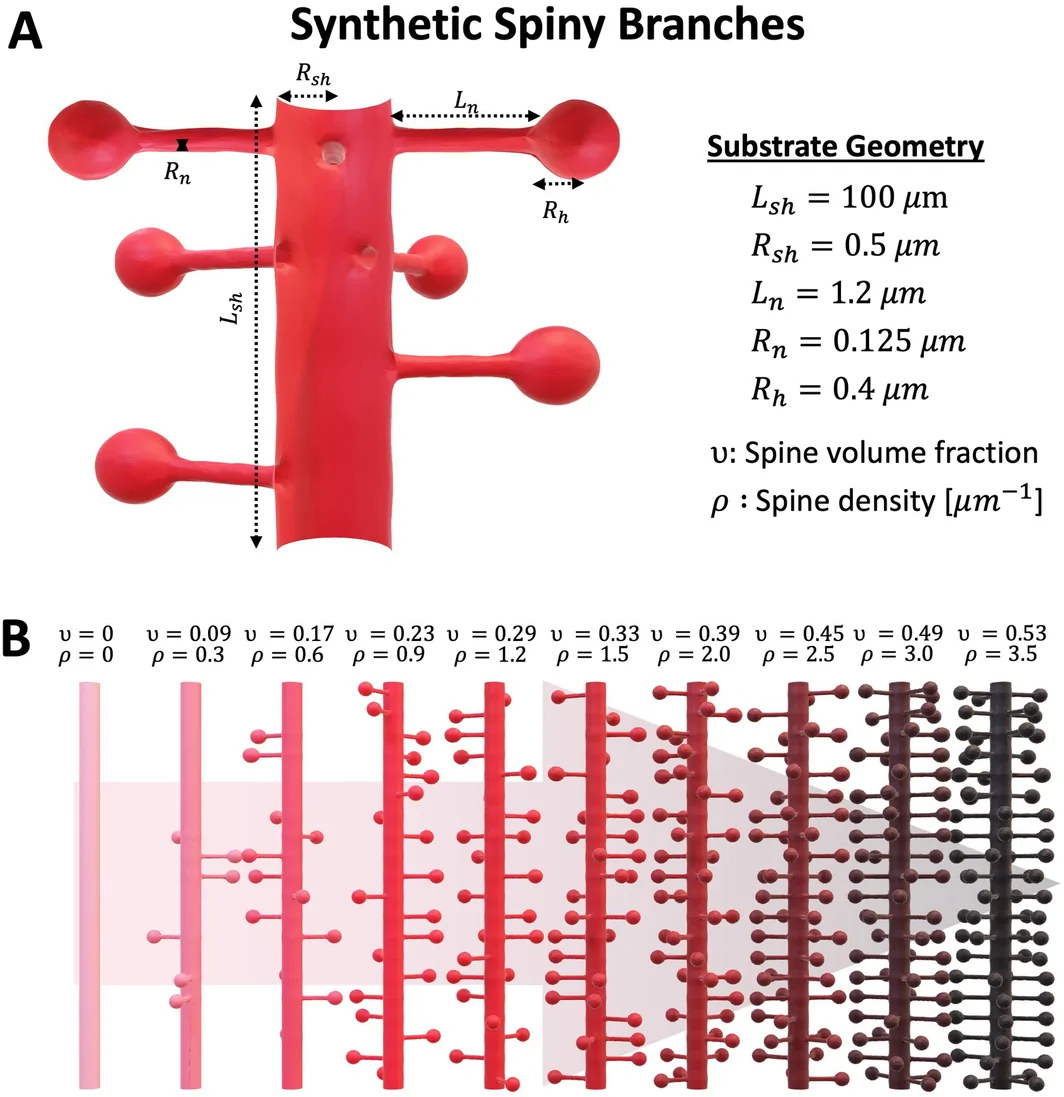
(A) An exemplary toy model for spiny dendritic branches. The substrate geometry was determined by the mean values reported in the literature. (B) Toy substrate models for dendritic branches are shown for increasing spine densities (ρ) and the corresponding spine volume fractions (ν) are also documented.

Skeletons of spiny dendritic branches were built on MathWorks MATLAB 2022a [120] involving functions from the Trees‐Toolbox [121]. The generated skeletons were exported as SWC file format for surface meshing in Blender API v4.1 using metaballs and the Blender addon publicly available at https://github.com/kdrsimsek/SWC_Mesher_v4x. The metaballs employed in 3D reconstruction of substrates were scaled to match the desired morphological sizes in each substrate. The 3D reconstructed spiny dendritic branches were further processed for cleaning from any mesh artifacts. Due to the surface meshing technique, the nominal sizes in the dendritic branches are slightly different from the prescribed skeleton ones. The dendritic spine sizes were based on the average values reported in the literature (see Section 2.1). The dendritic shaft geometry was defined by a length $L_{\mathrm{sh}}=100\;\mathrm{\mu m}$ and a radius $R_{\mathrm{sh}}=0.5\;\mathrm{\mu m}$. The spine geometry was specified by a head radius $R_{h}=0.4\;\mathrm{\mu m}$, while spine neck sizes were set to a length $L_{n}=1.2\;\mathrm{\mu m}$ and a radius $R_{n}=0.125\;\mathrm{\mu m}$. The resulting substrate models for the spiny dendritic branches are illustrated in detail in Figure 3, providing morphological information on the substrates.

Two sets of spiny dendritic segments were generated to investigate the exchange effect of diffusion in spines in the case of simple straight shaft and in the case of shaft with additional complexity, introducing undulation and/or beading. Our aim in generating the first set of branches was to study the impact of different spine densities (and spine volume fraction) on the diffusion exchange mechanism, without additional confounders stemming from shaft undulation and beading. Therefore, 10 spiny branches with typical spine densities ($\rho =\left[0-3.5\right]\;\mu m^{-1}$) were generated [122, 123]. The second set of spiny dendritic segments was generated to study the contributions stemming from other morphological features, that is, beading and/or undulation. The shaft was defined by a skeleton of connected nodes; each assigned a radius. For a smooth shaft, all nodes were given the same radius, producing a straight geometry. To introduce undulations, the node positions were modulated along the $\hat{z}$ axis using a cosine function, with the number of oscillations controlled by $N_{\mathrm{und}}$. To add beading, node radii were sampled from a uniform distribution between $R_{\mathrm{sh}}$ and $R_{\mathrm{sh}}+A_{\text{bead}}$ and assigned along the skeleton. To avoid abrupt and unrealistic radius changes between adjacent nodes, the sampled radii were smoothed along the shaft, ensuring gradual transitions while preserving the intended overall distribution. A total of 32 spiny branches at $\rho =\left[1,2\right]\;\mu m^{-1}$ were generated using the combination of four beading amplitudes ($A_{\text{bead}}=\left[0,1,2,3\right]\;\mathrm{\mu m}$) and four undulation periods ($N_{\mathrm{und}}=\left[0,0.75,1.5,3\right]$, yielding wavelengths $\lambda_{\mathrm{und}}=\left[-,120,60,30\right]\;\mathrm{\mu m}$). In the beaded branches, the spine neck lengths $L_{\text{n}}$ were maintained to be around 1.2μm by accounting for the beading amplitudes. Notably, any of these features can be changed arbitrarily; here, we investigated realistic spine densities and realistic to extreme undulations and beading in dendritic branches. All generated digital substrates and the codes for data analysis will be available on https://github.com/kdrsimsek/dendriticSpines_exchange upon publication.

### 3.2 Monte Carlo Diffusion Simulations

**FIGURE 4:**
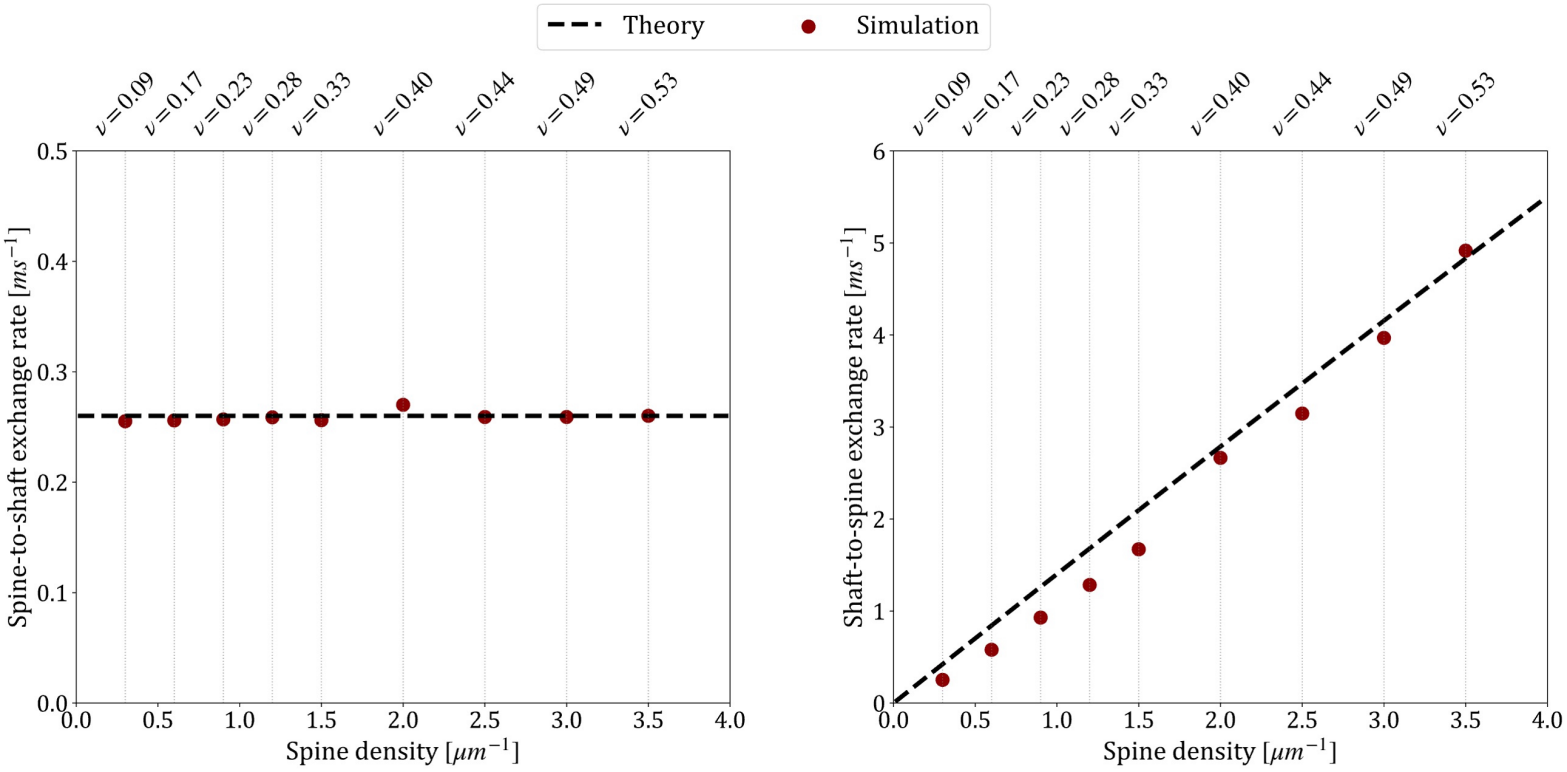
The comparison of estimated exchange rates from spine‐to‐shaft and shaft‐to‐spine with the theoretical predictions. The simulated $N\left(t\right)$ for each compartment fitted exponentially to estimate the exchange rates for different spine densities. The estimated exchange rates agree with the analytical solutions in Equations (1) and (2) for different spine densities.

The Python library disimpy [124] was used for Monte Carlo diffusion simulations. A few types of simulations were performed in this study, with the diffusivity set to the measured value for intraneurite water molecules in dMRI experiments [5, 13, 125], $2\;\frac{\mu m^{2}}{\mathrm{ms}}$, and periodic boundary conditions used for all diffusion simulations. The number of spins $N_{s}$ and the number of time steps $N_{t}$ in all simulations were determined by the Monte Carlo convergence method [126, 127]. The first set was used to generate particle concentrations within the spine and shaft, which were then used to analyze particle dynamics in the synthetic spiny branches and to tune $N_{t}$, ensuring that the particle behavior remained consistent with theoretical predictions. In this set, $N_{s}=10^{6}$ spins were simulated with a time step of 100 ns to record particle dynamics. The spins were initialized separately in the dendritic spines and the shaft to analyze their individual contributions to the particle dynamics. The time‐dependence of the number of spins inside dendritic spines (the concentration curve $N\left(t\right)$) was computed at each time step during Monte Carlo simulations. Then, $N\left(t\right)$ was determined, excluding any particles exiting the dendritic spines. To eliminate bias due to particle density within the dendritic spines, the initial number of particles at t=0 was set to be $N_{s}=10^{6}$ for each substrate. Consequently, the spine‐to‐shaft and shaft‐to‐spine diffusive exit rates were characterized by fitting the normalized $N\left(t\right)s$ to an exponential for $\frac{N\left(t\right)}{N\left(0\right)}\geq 0.5$ for maintaining balanced concentration during passage. Figure 4 shows the comparison between simulated exchange rates and theoretical predictions for both diffusive exit rates. The normalized concentration curves $\frac{N\left(t\right)}{N\left(0\right)}$ for the corresponding particle dynamics are shown in Figure S1.

After optimizing the particle dynamics of the tuned substrates, the second set of simulations was configured to have $N_{s}=10^{6}$ spins and a time step of 100 ns ($N_{t}$ was varied with the total simulation time) to generate diffusion signals. These simulations were designed to investigate the impact of dendritic spines on the diffusion exchange mechanism. Ten linearly spaced *b* values up to 7 $\frac{\mathrm{ms}}{\mu m^{2}}$ were employed, with diffusion gradients applied parallel ($\hat{z}$) and perpendicular ($\hat{x}$ and $\hat{y}$) to the dendritic shaft (oriented along $\hat{z}$), yielding $S_{∥}$ and $S_{⊥}$, respectively [52, 53]. Powder‐averaged signals ($S_{\mathrm{avg}}$) were generated using 128 uniformly distributed directions. A total of 12 different gradient schemes were constructed by trapezoidal gradients with fixed gradient ramp time 1 ms. The schemes were generated from combination of seven diffusion gradient separation times ($\Delta =\left[5,10,25,35,55,85,160\right]\;\mathrm{ms}$), with two gradient durations ($\delta =\left[3,15\right]\;\mathrm{ms}$, i.e., narrow and wide gradient pulses, respectively). Additional high *b* value regime simulations ($b=\left[3-20\right]\frac{\mathrm{ms}}{\mu m^{2}}$) were performed to study diffusion signal characteristics for $t_{d}s<60\;\mathrm{ms}$ using both pulse schemes. These parameters were tailored for preclinical scanners employing ultra‐strong gradients (i.e., $\geq 300\;\frac{\mathrm{mT}}{m}$).

The final set of Monte Carlo simulations was performed to investigate the time‐dependence of diffusivity and kurtosis within spiny dendritic branches, using $N_{s}=10^{6}$ spins and a time step of 100 ns. Apparent diffusion coefficients (ADC) and kurtosis ($K^{\mathrm{app}}$) were computed for all diffusion times using the cumulant expansion framework [21]. These simulations allowed us to study exclusively the time‐dependence of parallel and perpendicular diffusivity and kurtosis in the spiny branches.

**FIGURE 5:**
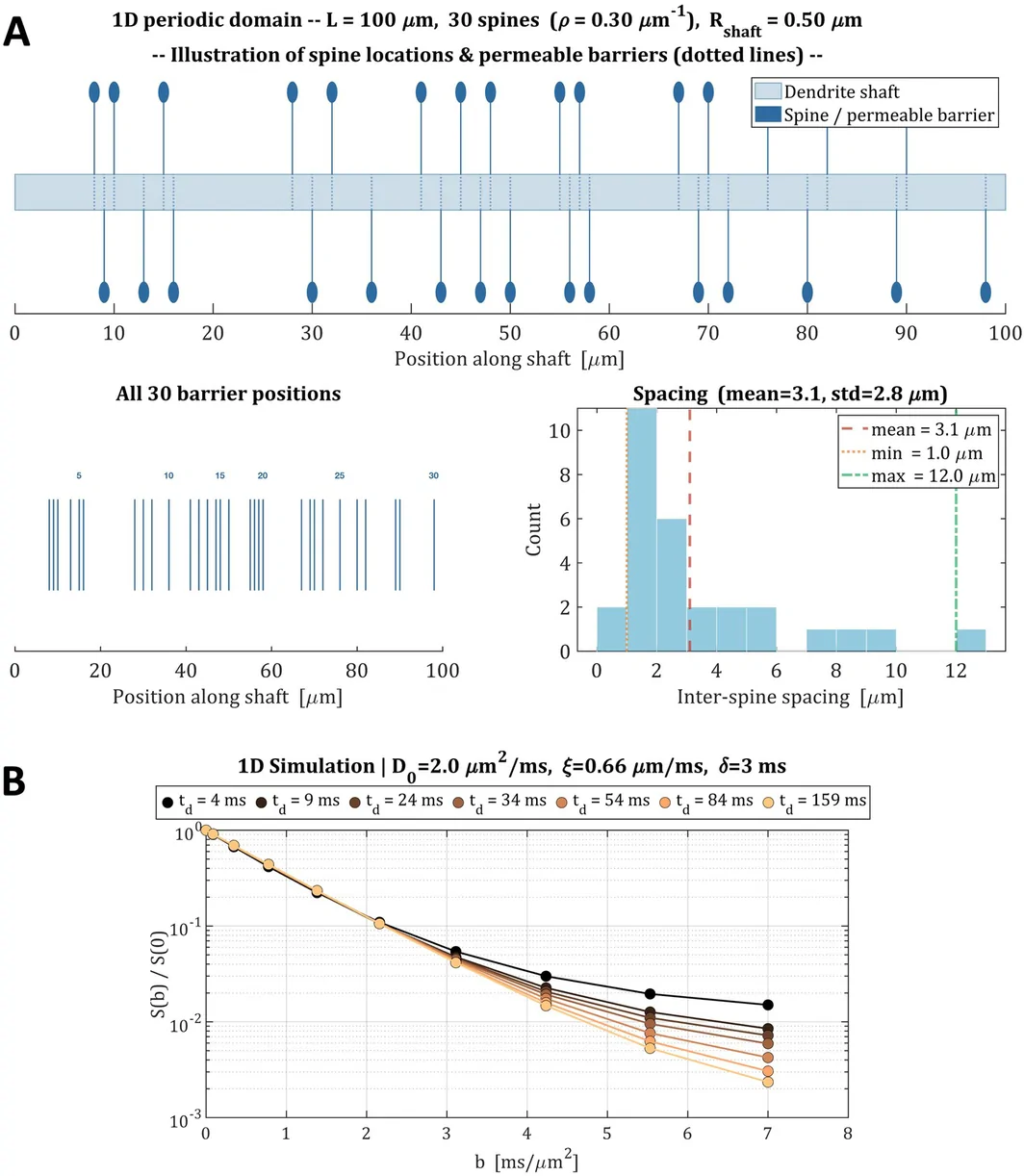
(A) Illustration of the equivalent 1D permeable‐membrane model. Membranes were positioned at locations corresponding to randomly distributed spine attachment sites along the dendritic branch. The histogram shows the resulting inter‐membrane spacing distribution for a spine density of $\left(\rho =0.3\;\mu m^{-1}\right),$ with a mean spacing of 3.1 μm. (B) Simulated diffusion‐weighted signals obtained from the 1D permeable‐membrane model using the narrow‐pulse encoding scheme.

To aid interpretation of the diffusion signal characteristics observed in spiny dendritic branches, we performed additional Monte Carlo simulations in a one‐dimensional permeable‐membrane geometry. Membranes were positioned at locations corresponding to spine attachment sites in branches with spine densities of ρ=0.3 and $0.6\;\mu m^{‐1}$ (Figure 5; see Figure S2 for $\rho =0.6\;\mu m^{-1}$). The resulting membrane spacing distribution reproduced the random spine placement along the dendritic branch, with a mean inter‐membrane distance of approximately 3 μm. Simulation parameters were matched to those used by Novikov et al. [17], including a membrane permeability of ($\xi =0.66\frac{\mathrm{\mu m}}{\mathrm{ms}}$). Diffusion‐weighted signals were generated using the narrow‐pulse encoding scheme.

### 3.3 Data Analysis

#### 3.3.1 Diffusion, Restriction and Exchange Analysis in Dendritic Spines

First, we examined whether the modified two‐compartment Kärger model (Equation (5)) could accurately describe $S_{∥}$. We fitted Equation (6) to estimate the exchange rates $k_{\mathrm{sp}\to \mathrm{sh}}$ and compare them with the theoretical predictions from Equation (1). Then, we used the measured volume fractions in our digital substrates to characterize the diffusion‐mediated exchange rate $k_{\mathrm{ex}}^{\mathrm{DM}}$ within the spiny dendritic branches and compared it to corresponding theoretical predictions for both pulse schemes.

In addition, we inspected the influence of dendritic shaft morphology by using synthetic spiny substrates with varying levels of undulation and beading. We estimated the exchange times using the modified Kärger model for these different undulation and beading levels to explore the impact of dendritic shaft morphology on exchange time estimations.

We analyzed the time dependence of $ADC_{∥}$, and $K_{∥}^{\mathrm{app}}$ along the dendritic shaft. Using linear regression, we fitted $ADC_{∥}$ and $K_{∥}^{\mathrm{app}}$ as functions of $t^{-\frac{1}{2}}$ (structural disorder) and $t^{-1}$ (exchange) and the simulated diffusion signals as a function of $b^{-\frac{1}{2}}$ (structural disorder) and $b^{-\frac{3}{2}}$ (exchange) to determine which mechanism between structural disorder and exchange dominates diffusion along spiny dendrites. Quantitatively, we compared goodness‐of‐fit *χ* ^2^ and Akaike information criterion (AIC) to determine which model better described the simulated trends.

In addition, we investigated the time dependence of $ADC_{⊥}$, perpendicular to the dendritic shaft, in the context of restriction. Specifically, we analyzed how dendritic spines modulate effective transversal displacement and how this modulation affects $\mathrm{AD}C_{⊥}$ and how it influences exchange measurements.

#### 3.3.2 Impact of Dendritic Spines on Time‐Dependent SDE Measurements and NEXI Analysis

To evaluate how diffusion‐mediated exchange between dendritic shaft and spines influences exchange rate estimates in NEXI analysis, we conducted two experiments. First, we simulated time‐dependent SDE signals using our synthetic substrates of spiny dendrites in extracellular space (with impermeable membranes) and fitted NEXI to assess how spine density affects permeative exchange rate estimates. Second, we incorporated membrane permeability using our extended three‐compartment Kärger model as forward model to examine how the combined effects of permeative and diffusion‐mediated exchange alter these estimates.

For the first experiment, to replicate diffusion signal from a dMRI voxel and compare it with NEXI biophysical modelling of in vivo experiments, we simulated the total signal $S_{\text{total}}$ arising from a dMRI voxel as the weighted sum of nonexchanging intraneurite and extraneurite signals, $S_{\text{neurite}}$ and $S_{\text{Gaussian}}$, respectively: $S_{\text{total}}=0.75\times S_{\text{neurite}}+0.25\times S_{\text{Gaussian}}$. The $S_{\text{neurite}}$ was derived from our simulations in spiny dendrites at different spine densities (with and without undulations and/or beadings) while $S_{\text{Gaussian}}$ is mono‐exponential decay with diffusivity of $1.0\frac{\mu m^{2}}{\mathrm{ms}}$. $S_{\text{total}}$ was fitted using the NEXI model to estimate the exchange rate ($k_{\mathrm{ex}}^{\text{NEXI}}$) for all simulated conditions.

For the second experiment, to investigate the contributions of exchange mechanisms (i.e., dendritic spines and permeative exchange), we used numerical solutions of the three compartment Kärger model (Equation 7a defined in Section 2.5) to generate diffusion signals and simulate the powder‐averaged signal (expected from a millimeter‐size dMRI voxel) by computing numerically their integral over η in Equation (4). Then, we fitted the NEXI model to the simulated signals to investigate how the estimated exchange rates estimated from NEXI are biased by the presence of dendritic spines. We used characteristic membrane permeability values ($\xi =\left[1-20\right]\times 10^{-3}\frac{\mathrm{\mu m}}{\mathrm{ms}}$) [14, 114] and typical total spine volume fractions ($\nu_{\mathrm{tot}}\leq 20\%$). The characteristic exchange rate from spine‐to‐shaft ($k_{\mathrm{sp}\to \mathrm{sh}}$) was computed by Equation (1) using the average values measured according to the heterogeneity of spines morphology in the literature and fixed to $k_{\mathrm{sp}\to \mathrm{sh}}=75.2\times 10^{-3}\;ms^{-1}$ [60, 74, 90, 117, 128]; the characteristic exchange rates from shaft to extracellular space ($k_{\mathrm{sh}\to e}$) and from spine to extracellular space ($k_{\mathrm{sp}\to e}$) were computed by using the relation, k=SVξ, where SV is the surface‐to‐volume ratio of the shaft and spine, respectively for each spine volume fraction. The diffusivities of each compartment were $D_{\mathrm{sp}}=0\frac{\mu m^{2}}{\mathrm{ms}}$ for spines (i.e., still water or dot compartment), $D_{\text{in}}=2.0\frac{\mu m^{2}}{\mathrm{ms}}$ for the intraneurites and $D_{e}=1.0\frac{\mu m^{2}}{\mathrm{ms}}$ isotropic Gaussian extracellular space.

## 4 Results

### 4.1 Diffusion and Exchange Mechanism in Spiny Dendrites

**FIGURE 6:**
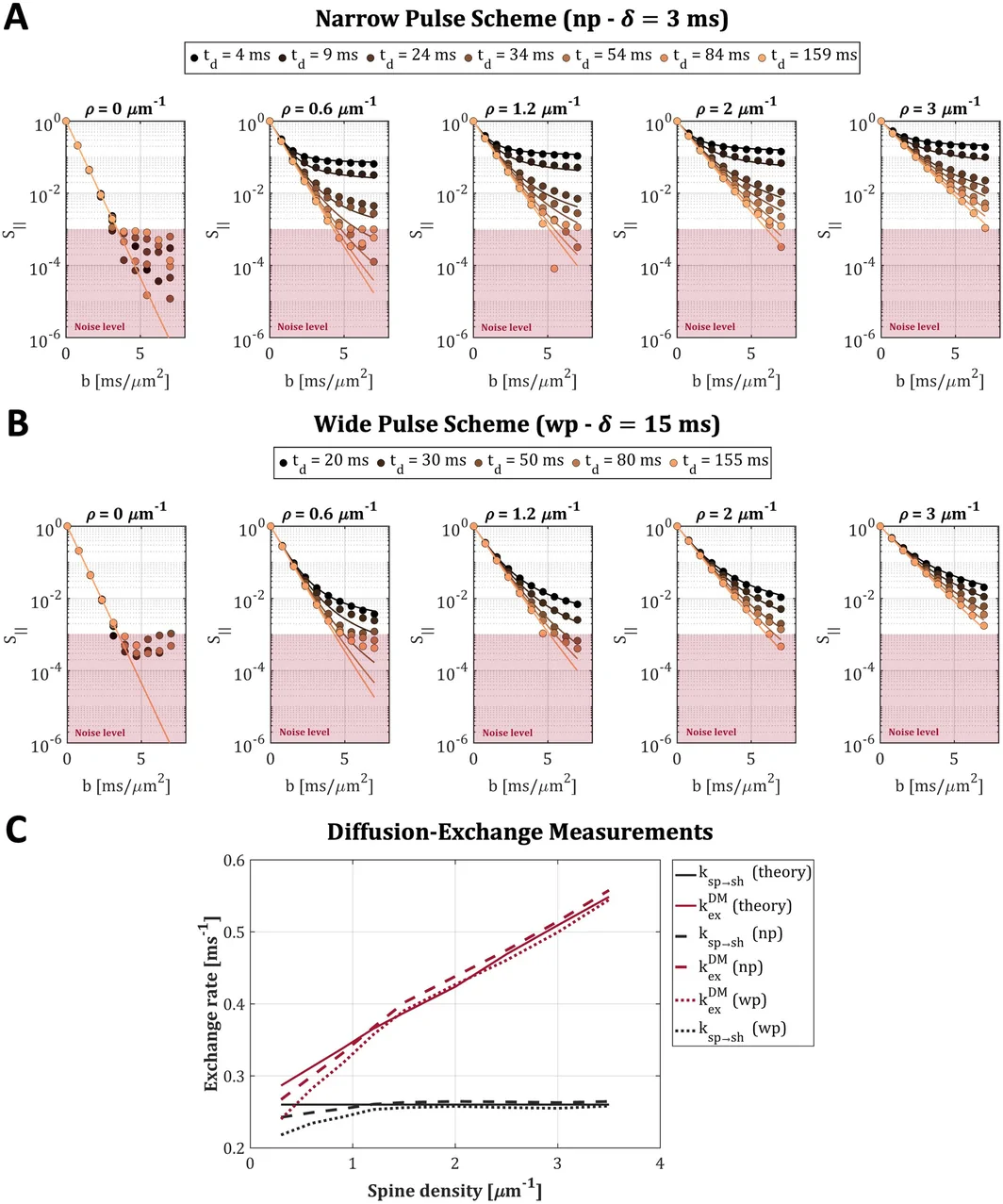
The simulated parallel diffusion signals ($S_{∥}$) are shown across all diffusion times using the narrow (A) and wide (B) pulse schemes. Solid lines in (A) and (B) present the fitted lines of the modified Kärger model. Briefly, two exchanging compartments (shaft and spines), where one compartment is modelled as fully restricted diffusion (the spines modelled as a Gaussian compartment with $D_{\mathrm{sp}}=0$). (C) The panel shows the estimated diffusive exit rate $k_{\mathrm{sp}\to \mathrm{sh}}$ (black) and diffusion‐mediated exchange rate $k_{\mathrm{ex}}$ (red) obtained using the modified Kärger model, for narrow (dashed) and wide (dotted) gradient pulses. These estimates are compared with the corresponding theoretical predictions ($k_{\mathrm{sp}\to \mathrm{sh}}$ and $k_{\mathrm{ex}}^{\mathrm{DM}}$; see Section 2.2). The dashed and dotted lines present the estimations of the modified Kärger model while the theoretical predictions are displayed as solid lines for both spine‐to‐shaft diffusive exit rates and the corresponding exchange rates. Both exchange rate estimates align with the theoretical predictions very well.

The simulated diffusion signals parallel to the dendritic shaft were fitted to the modified two‐compartment Kärger model. The fit results are shown in Figure 6A,B, together with the Monte Carlo simulated diffusion‐weighted signals parallel to the shaft main axis ($S_{∥}$) for all diffusion times, and for both narrow (A) and wide (B) pulse schemes. Figure 6C presents the estimated spine‐to‐shaft exit rates $k_{\mathrm{sp}\to \mathrm{sh}}$ (black) and the total diffusion‐mediated exchange rate of the system, $k_{\mathrm{ex}}^{\mathrm{DM}}$ (red), as functions of spine density for narrow (dashed) and wide (dotted) gradient pulses, alongside the theoretical predictions from Equation (1) (solid lines). Both estimated rates agree well with theory, except the low spine density regime exhibiting slight underestimations.

**FIGURE 7:**
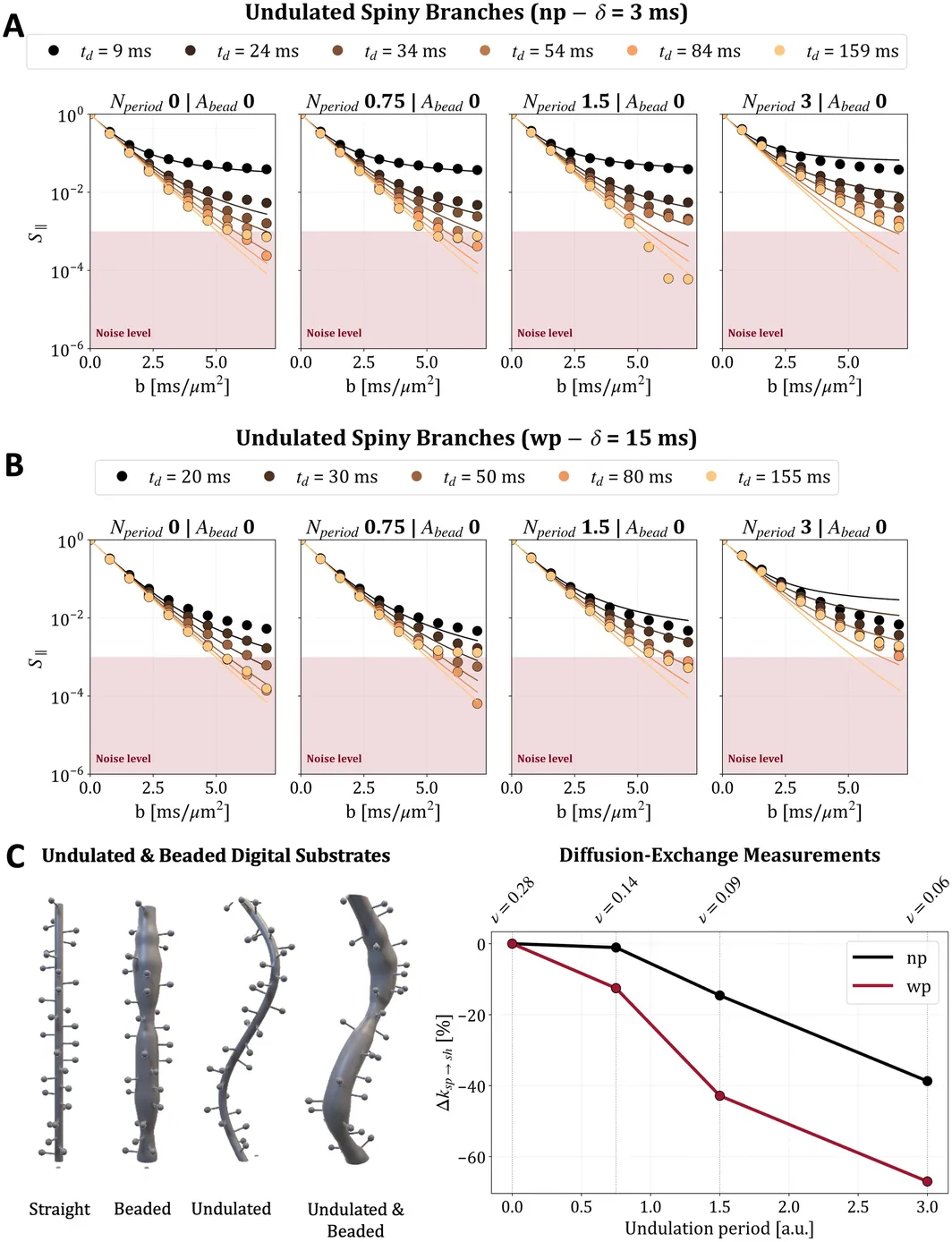
The diffusion signals in the parallel direction obtained from undulated substrates (no beading) with spine density $\rho =1\;\mu m^{-1}$ are shown in the figure for narrow (A) and wide (B) pulse schemes. At long diffusion times ($t_{d}>50\;\mathrm{ms}$), the restriction effect starts dominating the diffusion signal. In (C), the percentage change in the spine‐to‐shaft diffusive exit rate (${\mathrm{\Delta k}}_{\mathrm{sp}\to \mathrm{sh}}$), estimated by fitting diffusion signals to the modified Kärger model, is shown as a function of undulation period $N_{\mathrm{und}}$, relative to a reference branch without undulation or beading. For undulation period $N_{\mathrm{und}}<1.5$, the ${\mathrm{\Delta k}}_{\mathrm{sp}\to \mathrm{sh}}$ results present no impact of undulation on the estimated exit rates for the narrow pulse scheme and a small deviation (<15%) for the wide pulse scheme.

We studied the diffusion‐mediated exchange in more realistic spiny branches by introducing undulations and beading. As an exemplary illustration, the simulated diffusion signals in parallel direction within only undulated synthetic substrates at spine density of $\rho =1\;\mu m^{-1}$ are shown in Figure 7 for the narrow (A) and wide (B) gradient pulse schemes, respectively. Here, we focused on the results where we observed exchange signatures in our analysis. Figure S3 documents all simulated signals for $\rho =1\;\mu m^{-1}$ for both pulse schemes.

We fitted the modified Kärger model to the simulated signals to estimate $k_{\mathrm{sp}\to \mathrm{sh}}$ and computed the percentage change ${\Delta k}_{\mathrm{sp}\to \mathrm{sh}}$ relative to the estimates obtained from a substrate without undulation and beading ($k_{\mathrm{sp}\to \mathrm{sh}}≃0.27\;{\mathrm{ms}}^{-1}$). $\Delta k_{\mathrm{sp}\to \mathrm{sh}}$ as a function of undulation period $N_{\mathrm{und}}$ is reported in Figure 7C for narrow (black) and wide (red) pulses. For $N_{\mathrm{und}}<1.5$, no impact of undulation on $k_{\mathrm{sp}\to \mathrm{sh}}$ is observed for realistic undulation levels (${\Delta k}_{\mathrm{sp}\to \mathrm{sh}}\sim 0\%$) for narrow pulse while small deviation is observed in wide pulse scheme (${\Delta k}_{\mathrm{sp}\to \mathrm{sh}}<15\%$). However, for undulation periods $N_{\mathrm{und}}\geq 1.5$, significant underestimations (up to 40% for narrow and 65% for wide gradient pulses) are observed in the estimated $k_{\mathrm{sp}\to \mathrm{sh}}$ results. We only reported the impact of undulation as the modified Kärger model fits well only these signals, while it cannot characterize well the signals from substrates with beading. Because beading introduces additional restriction to the diffusion, it makes the signal restriction‐dominated and leads to a mismatch with the model. We also note that at long diffusion times ($t_{d}\geq 50\;\mathrm{ms}$), the effects of restriction become dominant in the diffusion signal, which deviates from the model predictions at high *b* values.

### 4.2 Diffusion Time‐Dependence in Spiny Dendritic Branches

**FIGURE 8:**
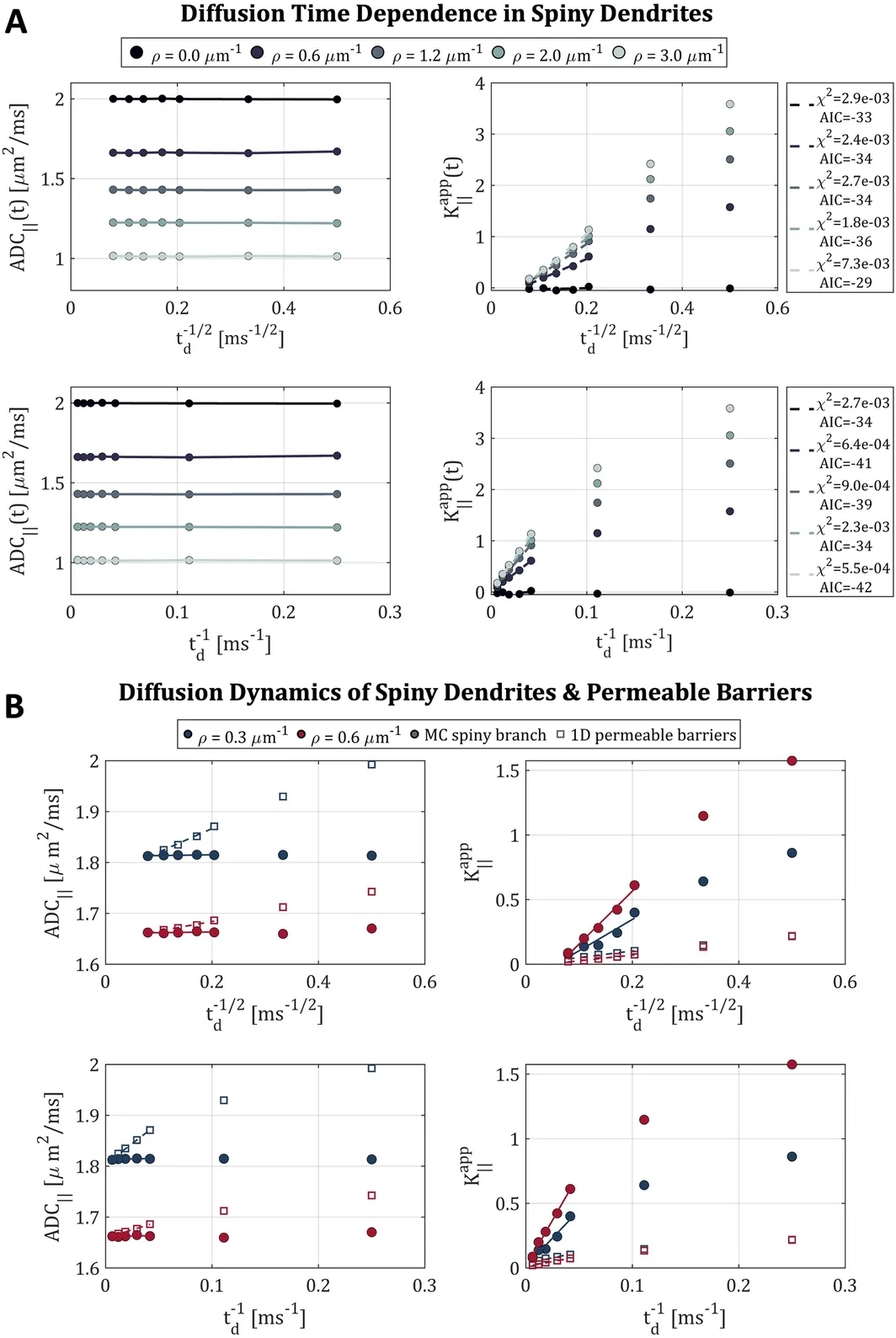
(A) The time‐dependence of the measured apparent diffusivities and kurtosis in directions parallel to the dendritic shaft is shown for several representative spine densities. The parallel diffusivity, ${\mathrm{ADC}}_{∥}$, shows no time‐dependence and decreases with increasing spine density, whereas the apparent kurtosis, $K_{∥}^{\mathrm{app}}$ is time dependent, with linear regression favoring the exchange signature ($t^{-1}$ over $t^{-\frac{1}{2}}$; *χ* ^2^ and AIC). (B) Comparison of $ADC_{∥}$ and $K_{∥}^{\mathrm{app}}$ estimated using the cumulant framework for spiny dendritic branches (circles) with spine densities of 0.3 (blue) and 0.6 (red) $\mu m^{‐1}$, and the corresponding 1D permeable‐membrane simulations (squares). For visual comparison, $ADC_{∥}$ from the 1D simulations was scaled to match the spiny branch values at the longest diffusion time. $K_{∥}^{\mathrm{app}}$ decreases with diffusion time in both models, consistent with exchange between compartments. In contrast, $ADC_{∥}$ remains nearly time‐independent in the spiny branch simulations, whereas the 1D permeable‐membrane model exhibits a progressive decrease with diffusion time, characteristic of one‐dimensional short‐range disorder [17].

To determine whether structural disorder or geometric exchange dominates diffusion within spiny dendritic branches, we examined the diffusion‐time dependence of the apparent axial diffusivity and kurtosis. $ADC_{∥}$ and $K_{∥}^{\mathrm{app}}$ are shown in Figure 8A against $t^{-\frac{1}{2}}$ and $t^{-1}$, the scaling signatures of structural disorder and exchange respectively. Over the investigated diffusion times, $\mathrm{AD}C_{∥}$ showed no time dependence but decreased with increasing spine density, whereas $K_{∥}^{\mathrm{app}}$ was clearly time dependent. Fitting both power laws to $K_{∥}^{\mathrm{app}}$, the goodness‐of‐fit metrics (χ^2^ and AIC) favored the $t^{-1}$ (exchange) scaling for all spine densities $\rho >0\;{\mathrm{\mu m}}^{-1}$ (Figure 8A), identifying exchange as the dominant mechanism in spiny branches.

To test whether spines act as one‐dimensional short‐range structural disorder along the dendrite, we simulated 1D diffusion across permeable barriers placed at the exact spine positions of branches, at two spine densities (*ρ* = 0.3 and $0.6\;\mu m^{‐1}$) using the identical narrow‐pulse scheme (Figure 5). Estimated $ADC_{\|}$ and $K_{∥}^{\mathrm{app}}$ of 1D permeable membrane simulations using the cumulant framework were compared with those from spiny dendrites simulations (Figure 8B). For visual comparison of the diffusion time‐dependence, $ADC_{\|}$ of 1D simulations was scaled by a constant factor to match the spiny dendrite $ADC_{∥}$ at the longest diffusion time. The barrier model failed to reproduce the spiny‐branch cumulants: it exhibited a time‐dependent $ADC_{∥}$, the signature of structural disorder ($t_{1}^{-\frac{1}{2}}$ scaling), whereas the spiny branch showed a time‐independent $ADC_{∥}$. Although both exhibited decreasing kurtosis with diffusion time, the signature of exchange ($t^{-1}$ scaling). Spines therefore do not behave as short‐range structural disorder; their diffusion signature is instead more consistent with exchange.

**FIGURE 9:**
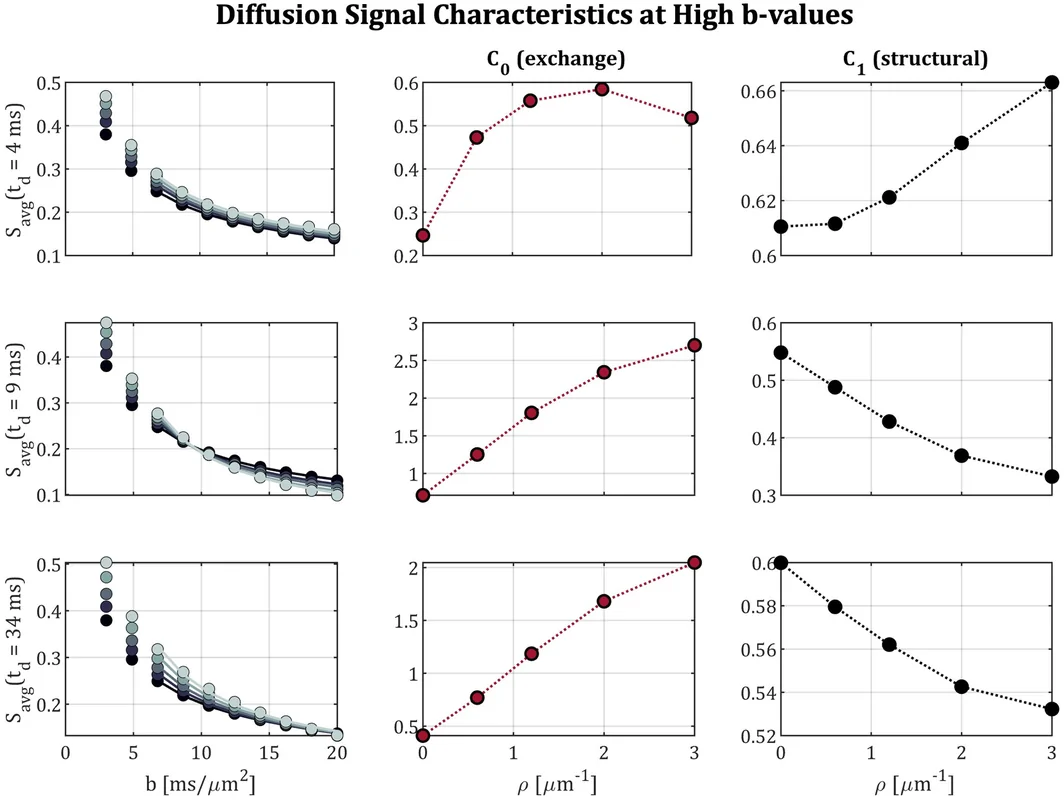
Powder‐averaged diffusion signals in high *b* value regime ($b=\left[3-20\right]\frac{\mathrm{ms}}{\mu m^{2}}$), fitted to the cubic parabola form $C_{0}b^{-\frac{3}{2}}+C_{1}b^{-\frac{1}{2}}$ for several spine densities and diffusion times. The fitted coefficients are plotted against spine density: the exchange coefficient $C_{0}$ increases with spine densities for all $t_{d}$s, while the structural‐disorder coefficient $C_{1}$ predominantly decreases. Together these analyses indicate that exchange is the dominant signature in spiny dendrites.

We further characterized the powder‐averaged signal $S_{\mathrm{avg}}$ in the high‐b regime by fitting a cubic‐parabola form, $C_{0}\cdot b^{-\frac{3}{2}}+C_{1}\cdot b^{-\frac{1}{2}}$, to the signal at $b>5\frac{\mathrm{ms}}{\mu m^{2}}$ (Figure 9; see all simulated diffusion signals in Figure S4), where this asymptotic expansion separates the exchange‐sensitive ($b^{-\frac{3}{2}}$) and structural‐disorder ($b^{-\frac{1}{2}}$) signatures. The two signatures showed opposing dependence on spine density: for $t_{d}\geq 9$ ms, the exchange coefficient $C_{0}$ increased with spine density while the structural coefficient $C_{1}$ decreased, identifying exchange as the dominant signal feature in spine‐rich substrate. At the shortest diffusion time ($t_{d}=4\;\mathrm{ms}$) this separation broke down—both coefficients increased with spine density—so neither signature could be isolated as dominant.

### 4.3 Restriction in Spiny Dendritic Branches

**FIGURE 10:**
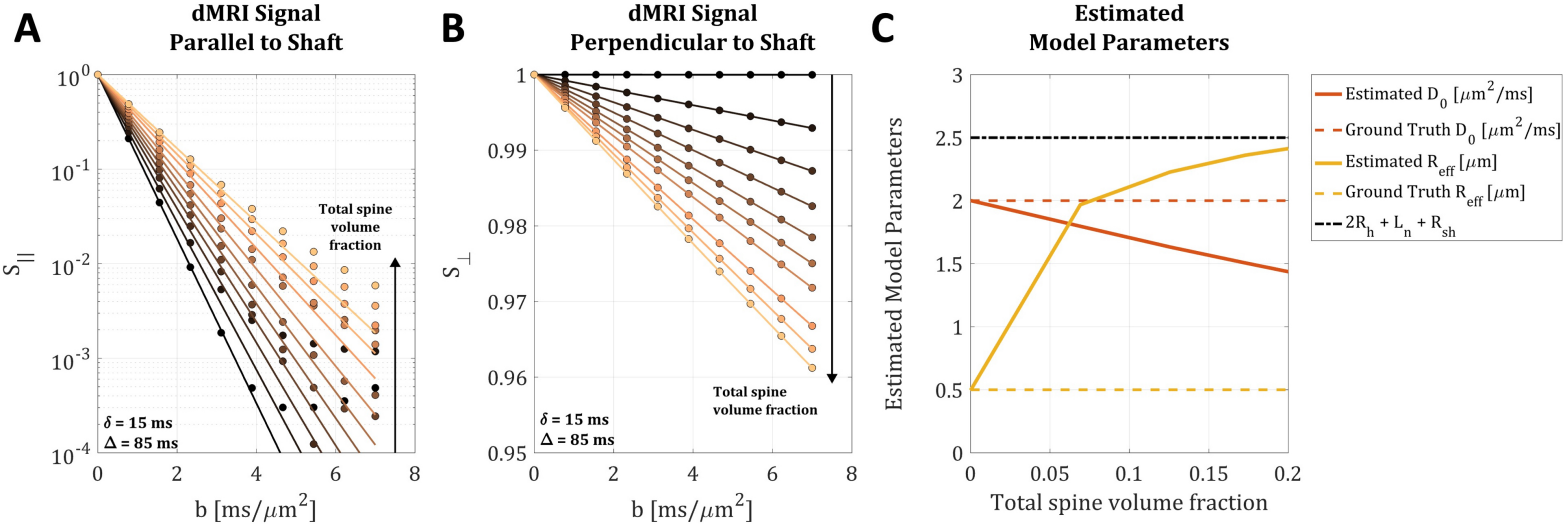
Simulated diffusion signals in parallel (A) and perpendicular (B) directions are shown for wide pulse scheme at $t_{d}=80\;\mathrm{ms}$. Arrows indicate the increasing trend for the total spine volume fraction. Restriction imposed by the spines has opposite characteristics; restriction increases in parallel direction with increasing total spine volume fraction (or density) while diffusion becomes less restrictive in transverse direction with increasing total spine volume fraction. (C) Estimated model parameters (solid lines, obtained from simultaneous modelling of parallel and perpendicular diffusion signals with randomly oriented cylinders in Neuman approximation [115, 129]; see Section 2.6) are compared with ground truth values (dashed). Measurable changes in $D_{0}$ and $R_{\mathrm{eff}}$ are observed within typical total spine volume fractions. The effective radius approximates to the maximum radius in spiny dendritic branches (dashed black line) with increasing total spine volume fraction.

We further analyzed the perpendicular component of the simulated diffusion signal ($S_{⊥}$) in spiny dendritic branches to investigate how well our coarse‐grained version of the three compartments Kärger model describes the whole system. Figure 10 illustrates the parallel and perpendicular signals simulated with δ=15ms and Δ=85ms (see Figure S6 for all signals), together with corresponding model parameter estimates obtained using the coarse‐grained extended Kärger model (see Section 2.6). As the total spine volume fraction increases, the parallel signal ($S_{∥}$) becomes more restricted, while the perpendicular signal ($S_{⊥}$) shows stronger attenuation (i.e., less restriction). This reflects the opposing effects of dendritic spines: they hinder diffusion along the shaft but increase the effective space for diffusion in the transverse direction by enlarging the apparent shaft radius. Although signal attenuation in the transverse direction is minimal under these diffusion conditions, the estimated model parameters still exhibit measurable changes in the presence of dendritic spines.

Specifically, within biological total spine volume fraction ranges, the estimated $D_{0}$ decreases while $R_{\mathrm{eff}}$ increases drastically with increasing total spine volume fraction and approaching the maximum available transverse compartment size ($R_{\max}=2R_{h}+L_{n}+R_{\mathrm{sh}}$), consistent with the theoretical predictions. These results were further validated by an orthogonal analysis. Estimating the effective radius from $\mathrm{AD}C_{⊥}\left(t\right)∼\frac{R_{\mathrm{eff}}^{2}}{4t}$ at long diffusion time shows the same $R_{\mathrm{eff}}$ increase with spine density (see Figure S7).

### 4.4 Impact of Dendritic Spines on Time‐Dependent SDE Measurements and NEXI Analysis

**FIGURE 11:**
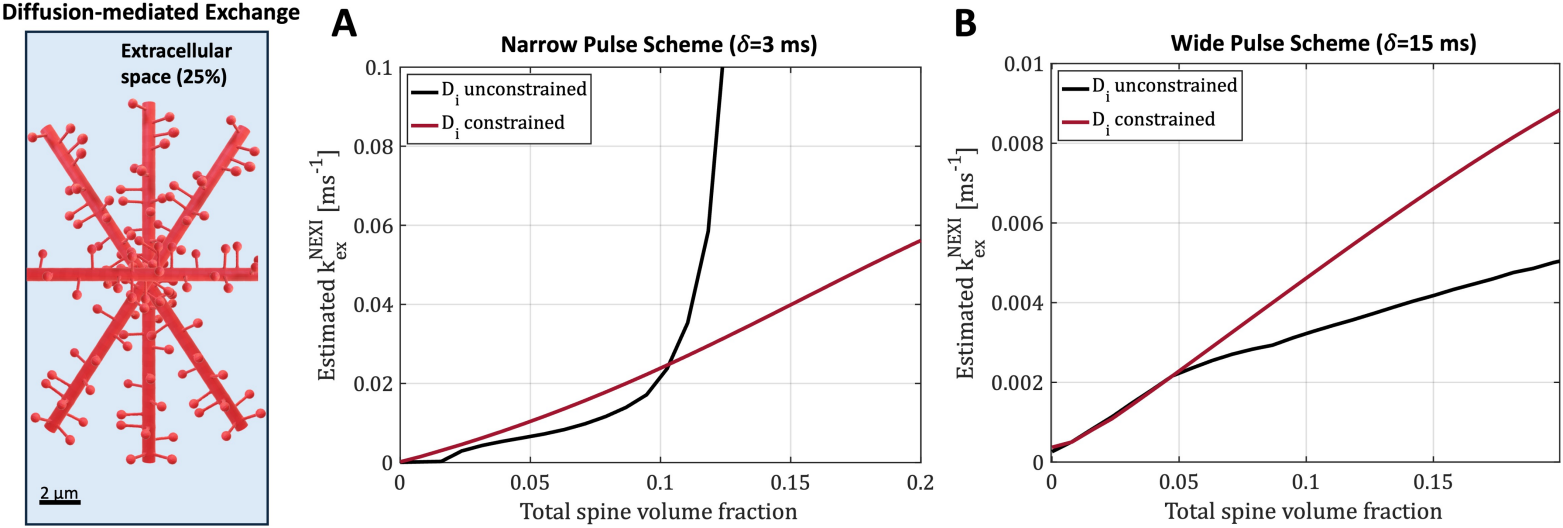
Exchange measurements in nonpermeable spiny branches (ξ=0). Bias in NEXI estimates of $k_{\mathrm{ex}}^{\text{NEXI}}$ is depicted by the diffusion‐mediated exchange within spiny dendritic branches for narrow and wide gradient schemes (black lines) when there is no permeative exchange between intracellular and extracellular compartments ($\xi =0\;\frac{\mathrm{\mu m}}{\mathrm{ms}}$). We generated the signal using 75% spiny dendrite +25% isotropic Gaussian extracellular space with diffusivity $1.0\frac{\mu m^{2}}{\mathrm{ms}}$ and fitted the NEXI model to it for different spine densities. The results are smoothed with a one‐point window for better visualization. The red lines correspond to a model fitting constrained by true axial intraneurite diffusivity $D_{i}$ to isolate the impact of dendritic spines. Despite the informed $D_{i}$, the exchange estimates still depend on the total spine volume fraction.

Figure 11 illustrates the diffusion‐mediated exchange system for the synthetic substrates without membrane permeability, incorporating a Gaussian extracellular compartment with a volume fraction of 0.25. The estimated exchange rates ($k_{\mathrm{ex}}^{\text{NEXI}}$) obtained from fitting the NEXI model to the total diffusion signal ($S_{\text{total}}=0.75\times S_{\text{neurite}}+0.25\times S_{\text{Gaussian}}$) are illustrated as a function of total spine volume fraction for both narrow (A) and wide (B) gradient pulse schemes. In the narrow pulse simulation results, the estimated exchange rates $k_{\mathrm{ex}}^{\text{NEXI}}$ varies between 0.01 and $0.1\;ms^{-1}$ for typical total dendritic spine volume fractions ($0.05\leq \nu_{\mathrm{tot}}\leq 0.2$ [114]) for narrow pulse simulations, whereas $k_{\mathrm{ex}}^{\text{NEXI}}$ estimations in the wide pulse acquisitions are drastically lower ($k_{\mathrm{ex}}^{\text{NEXI}}<0.01\;ms^{-1}$). For both schemes, we observe a spurious exchange effect when there is no permeative exchange between intra and extracellular domains, indicating a bias in exchange measurements in spiny dendritic branches.

The red lines in Figure 11 correspond to a diffusivity informed NEXI model fitting to “correct” bias in spiny dendritic branches. Intra axial diffusivities in spiny branches were directly measured from particle displacements for all spine densities (Figure S5) and subsequently fixed for the NEXI model fitting at typical $\nu_{\mathrm{tot}}\leq 0.2$. Figure 11 demonstrates that even if we would correct the intra axial diffusivity to account for the impact of dendritic spines, the bias in exchange estimates is not nulled, and a clear dependence on $\nu_{\mathrm{tot}}$ persists. Notably, fit results present more stable and consistent increase of $k_{\mathrm{ex}}^{\text{NEXI}}$ in both pulse schemes.

**FIGURE 12:**
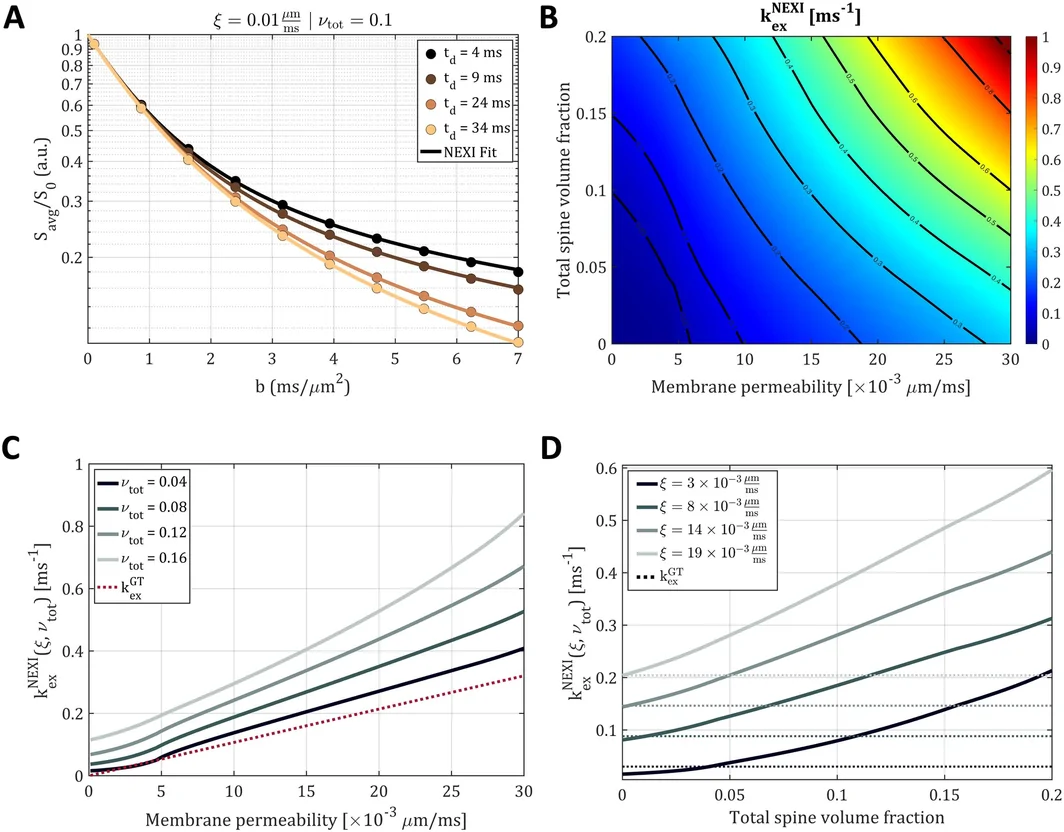
(A) Simulated diffusion signals at different diffusion times ($t_{d}$) using three‐compartment extended Kärger model (data points) (spine, shaft and extracellular space) are fitted with the NEXI/SMEX model (solid lines) for chosen membrane permeability ($\xi =10^{-2}\frac{\mathrm{\mu m}}{\mathrm{ms}}$) and total spine volume fraction ($\nu_{\mathrm{tot}}=0.1$). The NEXI/SMEX model perfectly fits the generated extended Kärger model signals. (B) The estimated exchange rates ($k_{\mathrm{ex}}^{\text{NEXI}}$) are mapped as a function of the membrane permeability and total spine volume fraction. The black isocurves highlight the degeneracy in the exchange estimations and document the $k_{\mathrm{ex}}^{\text{NEXI}}$ value in ${\mathrm{ms}}^{-1}$. The exchange rates increase with both the total spine volume fraction and the membrane permeability. Panels (C) and (D) illustrate the change in $k_{\mathrm{ex}}^{\text{NEXI}}$ as a function of membrane permeability (ξ) for multiple total spine volume fractions ($\nu_{\mathrm{tot}}=\left[0.04,0.16\right]$), and as a function of total spine volume fractions ($\nu_{\mathrm{tot}}$) for several membrane permeability values ($\xi =\left[3,19\right]\times 10^{-3}\frac{\mathrm{\mu m}}{\mathrm{ms}}$), respectively. The panels quantify the contributions of ξ and $\nu_{\mathrm{tot}}$ to the exchange estimations. Dotted lines indicate ground truth exchange rates $k_{\mathrm{ex}}^{\mathrm{GT}}$ when spines are unaccounted for exemplary permeability values.

To assess the impact of both exchange mechanisms (i.e., permeative and diffusion‐mediated exchanges) on the exchange rate measurements, Figure 12 presents a sensitivity analysis of exchange rates as a function of membrane permeability and total spine volume fraction. As an illustration, Figure 12A depicts the fitting of the NEXI model to diffusion signals simulated at multiple diffusion times ($t_{d}\leq 34$ ms) with a membrane permeability of $\xi =10^{-2}\frac{\mathrm{\mu m}}{\mathrm{ms}}$ and a total spine volume fraction of $\nu_{\mathrm{tot}}=0.1$. The estimated exchange rates ($k_{\mathrm{ex}}^{\text{NEXI}}$) as a function of permeability and total spine volume fraction are shown in Figure 12B. The estimated exchange rates in our simulations vary between $0-1\;{\mathrm{ms}}^{-1}$ and increase with both increasing permeability and total spine volume fraction. The degeneracy bands are highlighted with isocurves (black lines) which report the corresponding exchange rate in $ms^{-1}$. Furthermore, the exchange rate $k_{\mathrm{ex}}^{\text{NEXI}}\left(\xi ,\nu_{\mathrm{tot}}\right)$ is shown as a function of membrane permeability (for $\nu_{\mathrm{tot}}=0.04-0.16$) in Figure 12C and of total spine volume fraction (for $\xi =3-19\times 10⁻3\;\frac{\mathrm{\mu m}}{\mathrm{ms}}$) in Figure 12D. $k_{\mathrm{ex}}^{\text{NEXI}}$ increases with both membrane permeability (Figure 12C) and total spine volume fraction (Figure 12D). At fixed spine fraction, $k_{\mathrm{ex}}^{\text{NEXI}}$ scales approximately linearly with permeability but retains a nonzero intercept at *ξ* = 0 that increases with $\nu_{\mathrm{tot}}$, from $\approx 0.016\;{\mathrm{ms}}^{‐1}$ at $\nu_{\mathrm{tot}}=0.04$ to $\approx 0.12\;{\mathrm{ms}}^{‐1}$ at $\nu_{\mathrm{tot}}=0.16$ (Figure 12C). This finite intercept reflects spine‐mediated exchange that persists in the absence of transmembrane permeability. At fixed nonzero permeability, $k_{\mathrm{ex}}^{\text{NEXI}}$ rises with spine fraction across all permeabilities tested, the curves remaining nonzero and separated by ξ throughout (Figure 12D). The spine‐free reference rate $k_{\mathrm{ex}}^{\mathrm{GT}}=\xi \cdot \frac{SV_{\mathrm{sh}}}{f}$ is overlaid in both panels (dotted): $k_{\mathrm{ex}}^{\text{NEXI}}$ exceeds $k_{\mathrm{ex}}^{\mathrm{GT}}$ throughout, and the gap widens with spine fraction, quantifying the overestimation of the apparent exchange rate—and hence of inferred membrane permeability—that arises when dendritic spines are not accounted for in the model. The fitted rate thus depends jointly on both parameters and cannot be attributed to either mechanism individually: increases in membrane permeability and/or spine density both enhance the estimated $k_{\mathrm{ex}}^{\text{NEXI}}$. Disentangling the individual contributions of membrane permeability and dendritic spines to the exchange mechanisms is nontrivial and remains degenerate when using time‐dependent SDE measurements.

## 5 Discussion

Diffusion in dendritic spines has been extensively studied in the literature. Before diffusion MR, interest in molecular diffusion within dendritic spines was pioneered in cellular biology, leading to the development of various diffusion models to better understand these processes [96, 97, 101, 130]. In the diffusion MR field, few studies focused on the impact of dendritic spines on the diffusion measurements. A study examined the metabolite diffusion in silico and in vivo in rodents to inspect the effects of dendritic spines on the diffusion signal using diffusion‐weighted MR spectroscopy [131]. This work focused on restriction and showed that the presence of dendritic spines leads to larger estimates of neurite size and SV from SDE data acquired with both pulsed gradients at high *b* value and oscillating gradients at high frequency. Following works further investigated the impact of dendritic spines on both metabolite and water diffusion signals [52, 53, 54, 132] and provided evidence supporting the hypothesis that it is possible to measure the impact of dendritic spines on the intracellular diffusion MR signal. Only some recent studies focused on the potential role of dendritic spines in diffusion exchange measurements [52, 54].

In this study, we present an in‐depth analysis of the potential role of dendritic spines in diffusion exchange measurements using SDE, extending and complementing prior research. We evaluate how well different biophysical models—based on the popular Kärger model—describe diffusion‐mediated exchange between dendritic shafts and spines, leveraging ad‐hoc Monte Carlo simulations and theoretical insights from the “narrow escape problem.” Furthermore, we assess the extent to which such diffusion‐mediated exchange biases permeative exchange estimates in time‐dependent SDE measurements when analyzed with NEXI modeling. Our findings highlight the importance of accounting for diffusion‐mediated exchange in SDE measurements, cautioning against interpretations based exclusively on permeative exchange mechanisms. Below, we discuss each of these contributions in detail.

### 5.1 Diffusion‐Mediated Exchange in Spiny Dendrites

Adapting “the narrow escape problem” to biophysical modelling of exchange in spiny dendrites seems feasible and agrees with the modified Kärger model of two exchanging compartments [12] (Figure 6). Considering the spine‐to‐shaft exchange, the expected exit rate of molecules from spines based on theoretical predictions [101, 102] agrees very well with the exchange rate estimations from the modified Kärger model [12] with narrow and wide pulse settings (Figure 6C). In addition, one of our major findings shows that the modified Kärger model Equation (6) accurately characterizes the diffusion signal within spiny dendrites and the corresponding intracellular time‐dependent SDE signal (Figure 6A,B). Using a wide pulse in the modified Kärger model better satisfies the dot compartment condition, which might explain better agreement of both $k_{\mathrm{ex}}$ measurements in Figure 6C with theory compared with the narrow pulse case. However, it is worth mentioning that there is some bias in the fit with narrow and wide pulses, at short diffusion times and low spine densities (Figure 6C). This is probably due to noise effects, which are particularly important in those cases where the signal decays faster. Nevertheless, the Kärger model assumes that the impact of exchange during diffusion encoding is negligible, and the probability of spins' membrane permeation is constant, that is, short gradient pulse approximation [1, 12, 19, 106]. Accordingly, the wide pulses in the SDE measurements, violating the model assumption, lead to underestimation in exchange rate estimations in the model fitting [14, 19, 43]. Therefore, we raise caution when interpreting the model predictions obtained from wide pulse measurements.

The diffusion‐mediated exchange between spine and shaft exhibits the time‐dependent signature of permeative exchange across cell membrane and yields exchange rate on the order of $0.1\;{\mathrm{ms}}^{-1}$ for typical total spine volume fractions ($\nu_{\mathrm{tot}}<0.2$) [114]. This estimate aligns with previously reported permeative exchange rates ($0.01‐0.2\;{\mathrm{ms}}^{‐1}$) for neurites with radii in the range of 0.25‐1 μm and membrane permeabilities $\xi =\left[1-20\right]\times 10^{-3}\frac{\mathrm{\mu m}}{\mathrm{ms}}$, surrounded by an extracellular space with a volume fraction of 0.25 [13, 14, 42]. Therefore, the two exchange mechanisms cannot be easily disentangled using time‐dependent SDE measurements. Using advanced diffusion encodings, for example, double diffusion encoding (DDE) and free gradient waveforms, might be needed to resolve the confounding effects of these mechanisms, as we propose in another study which complements the current one [55].

We further investigated relatively realistic spiny dendrites using beaded and undulated substrates. While the modified Kärger model accurately describes diffusion signals from straight spiny dendritic branches, it fails to capture the signals from beaded and undulated geometries due to additional restriction effects, unaccounted for by the model (Figure 7A,B). This finding is consistent with previous literature [22, 112]. Abdollahzadeh et al. recently demonstrated that axonal beadings introduce a universal one‐dimensional structural disorder (i.e., a $t^{-\frac{1}{2}}$ scaling), which slows diffusion within the beaded axons, arising from longitudinal variations in beading amplitude that act as effective axial scattering sites [22]. Notably, signals from purely undulated branches align with the model only up to a diffusion time $t_{d}\leq 50\;\mathrm{ms}$ as shown in Figure 7. For $N_{\mathrm{und}}<1.5$, the estimated spine‐to‐shaft exchange rate $k_{\mathrm{sp}\to \mathrm{sh}}$ is consistent with the theoretical predictions obtained for the narrow pulse scheme. In contrast, the wide pulse scheme exhibits deviations in $\Delta k_{\mathrm{sp}\to \mathrm{sh}}$ of up to 15%, likely reflecting an underestimation of the exchange rate [14, 19, 43]. For highly undulated branches ($N_{\mathrm{und}}\geq 1.5$ or $\lambda_{\mathrm{und}}\leq 60\;\mathrm{\mu m}$), the deviations increase substantially for both pulse schemes, suggesting that axial restriction effects become dominant.

### 5.2 Interplay Between Structural Disorder and Exchange in Spiny Dendritic Branches

We investigated the time‐dependence of the apparent parallel diffusivity ($ADC_{∥}$) and kurtosis ($K_{∥}^{\mathrm{app}}$), as well as the apparent perpendicular diffusivity ($ADC_{⊥}$) relative to the dendritic shaft, using cumulants [21] for narrow pulse scheme. For the investigated diffusion time range, the ${\mathrm{ADC}}_{∥}$ exhibits no time‐dependence and decreases with increasing spine density, consistent with increased axial hindrance due to spines. In contrast, Lee et al. identified structural disorder as the underlying mechanism modifying mean diffusion along dendritic shafts [18]. This difference might originate from the fact that we particularly focus on the parallel diffusivity instead of mean diffusivity.

To disentangle the interplay between exchange and structural disorder, we also studied $K_{∥}^{\mathrm{app}}\left(t\right)$ and found that $K_{∥}^{\mathrm{app}}$ decreases with diffusion time $t_{d}$, which agrees qualitatively with recent findings in cortical gray matter reported by Lee et al. for mean kurtosis [18]. Like the diffusivity comparison, while that study inferred a $t^{-1/2}$ scaling of mean kurtosis and attributed it to one‐dimensional short‐range structural disorder along neurites, it did not explicitly examine the time‐dependence of directional kurtosis components (i.e., parallel kurtosis). Figure 8A depicts $K_{∥}^{\mathrm{app}}$ as a function of $t^{-\frac{1}{2}}$ and $t^{-1}$ to visualize, respectively, the characteristic signatures of structural disorder and exchange. Linear regression of $K_{∥}^{\mathrm{app}}$ against both $t^{-\frac{1}{2}}$ (structural disorder) and $t^{-1}$(exchange) reveals that exchange signature exhibits better performance and therefore is the dominant mechanism (lower $\chi^{2}$ values and AIC).

We performed three distinct analyses to reveal the underlying signature of diffusion dynamics in spiny dendrites, all converging on exchange as the dominant diffusion mechanism. From the Monte Carlo simulations, the cumulant time‐dependence showed a flat $\mathrm{AD}C_{∥}$ and a kurtosis decaying as $t^{-1}$ rather than $t^{-\frac{1}{2}},$ AIC‐favoured at all nonzero spine densities (Figure 8A). Independently, a 1D permeable‐barrier model representing spines as short‐range structural disorder failed to reproduce the branch cumulants—exhibiting a time‐dependent diffusivity and weak kurtosis without the $t^{-1}$ scaling—confirming that spines do not act as 1D disorder (Figure 8B). Finally, the high‐b signal decomposition showed the exchange coefficient $C_{0}$ rising with spine density as the structural coefficient $C_{1}$ declined (Figure 9).

Taken together, our findings indicate that diffusion‐mediated exchange dominates diffusion process, while signatures of intrinsic 1D short‐range structural disorder play a secondary role and may become important at diffusion times ($t_{d}>34\;\mathrm{ms}$). Notably, this scope is specific to water; for 4–5× slower metabolite‐like dynamics, short‐range structural disorder has been shown to play a clearer and more measurable role [131].

### 5.3 Characterizing the Bias on Diffusion Exchange Estimates

The NEXI fit of the simulated dMRI signals for our system comprised of randomly oriented impermeable spiny dendrites, immersed in isotropic extracellular space, shows that the estimated exchange rate is not zero, and depends on the total volume fraction occupied by dendritic spines. This means that the time‐dependent signature induced by geometric exchange between spines and shaft has been captured by the NEXI's exchange rate parameter, even if there is no intraneurite/extraneurite exchange ongoing. Therefore, the exchange rate estimated by NEXI cannot be solely interpreted as permeative intracellular/extracellular exchange rate. Moreover, slower exchange rates are observed (i.e., overestimation of exchange time) for wide gradient pulses, in agreement with existing literature findings [13, 14, 42, 43].

One possible explanation for the spurious dependence of NEXI estimated exchange rate on spine density could be that the intra neurite axial diffusivity is not the same for each spine density but rather expected to decrease as spine density increases. To correct (or inform) for such bias induced by the dendritic spines and investigate whether this would eliminate the observed dependence of NEXI estimated exchange rate on spine density, we fixed the parallel diffusivities to the values computed from the spin dynamics for each spine density (or volume fraction) (see Figure S5), in the NEXI model fitting. By providing the true axial diffusivity to the NEXI model fitting, we estimated the exchange rates for both pulse schemes (red lines in Figure 11). However, the estimated exchange rates have remained dependent on the spine volume fraction, interestingly presenting a clear and monotonic trend with total spine volume fraction. This demonstrates that the exchange rate estimated by NEXI accounts for both geometric and permeative exchange mechanisms, in a nontrivial way and caution should be taken when interpreting NEXI exchange rate estimates solely in terms of permeative exchange.

Our simulations using an extended three‐compartment Kärger model further demonstrate that both diffusion‐mediated exchange between spines and shaft and membrane permeability significantly influence diffusion signals in GM. The NEXI model accurately fits these simulated signals (Figure 12A), with sensitivity maps revealing the degenerate nature of exchange rate estimates ($k_{\mathrm{ex}}^{\text{NEXI}}$) arising from both mechanisms (isocurves in Figure 12B). This degeneracy aligns with known limitations of NEXI/SMEX models in separating restriction from exchange effects [13, 14, 16, 44], cautioning against interpreting exchange rates purely as permeative membrane effects.

Furthermore, the model fitting of two‐compartment NEXI to signals from spiny dendrites systematically overestimates the apparent exchange rate. Due to the stick assumption in the NEXI model, the additional diffusion‐mediated exchange between dendritic shafts and spines is absorbed into the fitted rate. This bias has two distinct origins: a geometric, diffusion‐mediated component arising from shaft–spine exchange, which persists even in the absence of transmembrane permeability ($k_{\mathrm{ex}}^{\text{NEXI}}\approx 0.016-0.12\;{\mathrm{ms}}^{‐1}$ at ξ=0 across $\nu_{\mathrm{tot}}=0.04-0.16$; Figure 12C), and a transmembrane component that scales with permeability. Correspondingly, at fixed permeability the apparent rate increases monotonically with spine volume fraction (Figure 12D), so that tissues differing only in spine content would yield different $k_{\mathrm{ex}}^{\text{NEXI}}$ despite identical membrane properties. At low ξ and $\nu_{\mathrm{tot}}$ values the apparent rate can instead be underestimated due to insufficient time‐dependent contrast across diffusion time, which may also lead to fitting instabilities. Nevertheless, a single fitted exchange rate cannot separate these contributions; consequently, when spines are present, the apparent exchange rate cannot be interpreted as a direct measure of membrane permeability, and permeability inferred from the shaft surface‐to‐volume ratio is correspondingly overestimated. These results can help interpret variations in exchange rate measured in conditions or pathologies that are known to alter spine density. For example, in autism spectrum disorder, where spine density in temporal lobe deep layers is ∼20% higher than in controls ($\rho ∼1\;\mu m^{-1}$ versus $\rho ∼0.8\;\mu m^{-1}$; corresponding to $\nu_{\mathrm{tot}}∼0.17$ and $\nu_{\mathrm{tot}}∼0.14$, respectively [82]), our results suggest a corresponding ∼14% increase in $k_{\mathrm{ex}}^{\text{NEXI}}$ (at $\xi =0.01\frac{\mathrm{\mu m}}{\mathrm{ms}}$) that could be misattributed to increased permeability.

The exchange measurements from NEXI fitting at total spine volume fractions of 10%‐20% (∼10‐50 ms) align with previously reported in vivo human measurements [15, 42, 43]. Chan et al. [15] reported a cortical median exchange time of 13 ms, with lobar values ranging from ∼15 ms in frontal to ∼34 ms in occipital cortex, and attributed the faster frontal versus occipital exchange to cortical myelination. Spine density is higher in association cortex such as frontal than in primary sensory regions [69, 133], and our simulations show that higher spine volume fraction alone increases the apparent exchange rate. Because spine density and myelination co‐vary spatially—with cortical exchange rate recently shown in vivo to correlate positively with synaptic density and negatively with myelin as complementary drivers [134]—low spine volume fraction is a plausible additional factor to myelination resulting in slower exchange measurements.

**FIGURE 13:**
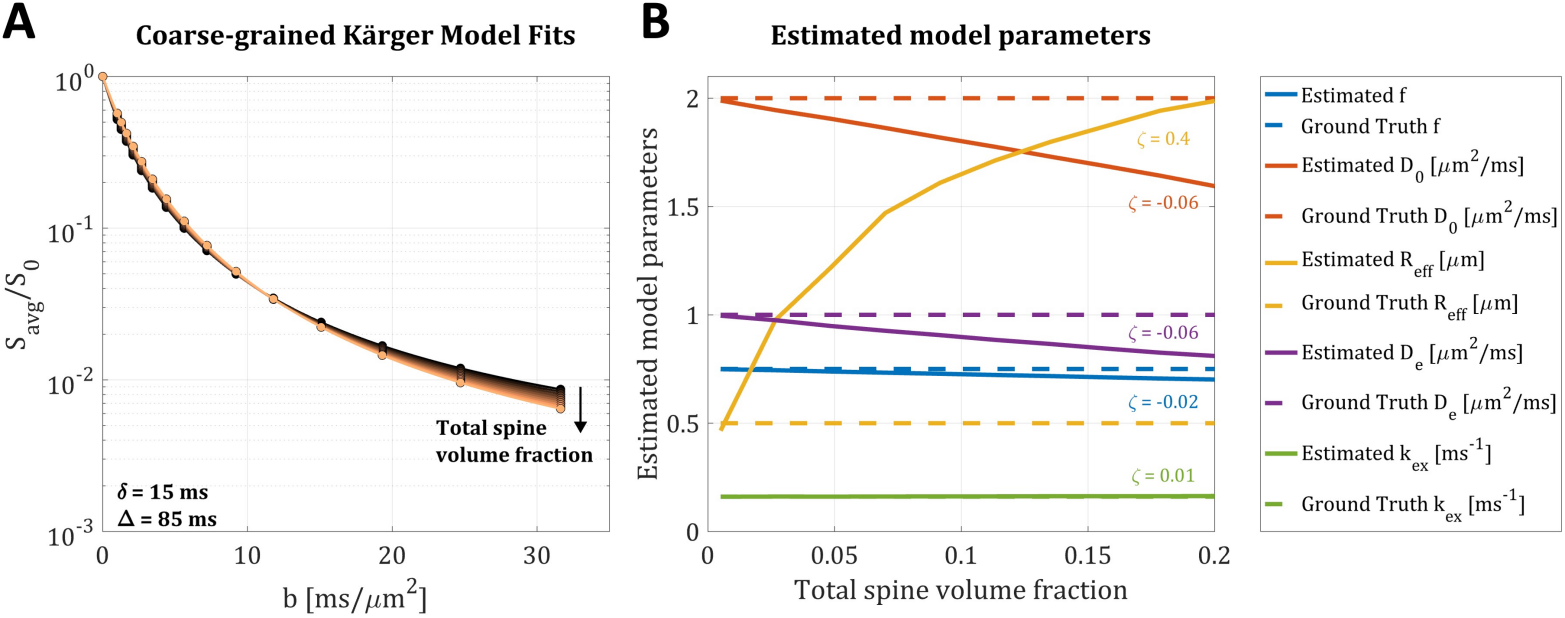
(A) Simulated diffusion signals at $t_{d}=80\;\mathrm{ms}$ were generated using three‐compartment extended Kärger model (data points), assuming biologically plausible membrane permeability ($\xi =10^{-2}\frac{\mathrm{\mu m}}{\mathrm{ms}}$) and a range of total spine volume fraction ($\nu_{\mathrm{tot}}=\left[0.005-0.2\right]$). The simulated signals are fitted with the coarse‐grained extended Kärger model (solid lines). (B) Estimated model parameters are shown as a function of $\nu_{\mathrm{tot}}$ and compared with the corresponding ground truth values. Elasticity parameter ζ quantifies measurable sensitivity in the model parameters, suggesting a strong dependence of $D_{0}$ and $R_{\mathrm{eff}}$ on the total spine volume fraction ($\left|\zeta \right|>0.05$).

In the motional narrowing regime, fine structures within a dMRI voxel are averaged out, making the signal sensitive to global microstructure [16, 110]. Here, dendritic spines can effectively turn “stick‐like” neurites into cylinders with an apparent radius, reducing the three‐compartment extended Kärger system to a two‐compartment model, that is, a ‘cylindrical’ NEXI. To test this, we simulated diffusion MRI signals with Δ=85ms (≈ 20× the typical intraspine residence time), δ=15ms, and $b\leq 30\frac{\;\mathrm{ms}}{\mu m^{2}}$ using our extended three‐compartments Kärger model (Figure 13A), fixing the membrane permeability to $10^{-2}\frac{\mathrm{\mu m}}{\mathrm{ms}}$ and ranging the total spine volume fraction from 0.5% to 20%, biologically plausible value(s). We then fitted our coarse‐grained extended Kärger model to these signals, and we quantified the sensitivity of each model parameter to spine volume fraction via the elasticity parameter $\zeta =\frac{\text{dlog}\left(\text{parameter}\right)}{\text{dlog}\left(\text{total spine volume fraction}\right)}$, as recently proposed by Jensen et al. [10]. We found that all the estimated parameters ($D_{0},R_{\mathrm{eff}},D_{e},k_{\mathrm{ex}},f$) varied with total spine volume fraction, confirming degeneracy between diffusion‐mediated and permeative transmembrane exchange (Figure 13B). However, $R_{\mathrm{eff}}$ showed the strongest measureable sensitivity (ζ=0.4); the diffusivities ($D_{0}$ and $D_{e}$) varied marginally (∣ζ∣>0.05). These results support the idea that a coarse‐grained extended Kärger model simplifies biophysical modelling, allowing diffusion‐MRI signatures of spine‐mediated exchange to be interpreted as “effective cylindrical” dendrites exchanging with the extracellular space.

Collectively, these findings underscore dendritic spines as a critical, though often overlooked, source of nonpermeative exchange. Alongside myelination and other microstructural factors [23], spine density variations likely drive the observed regional differences in exchange estimates across the brain [13, 14, 30, 43].

Finally, it is worth noting that dendritic spines can have a substantial impact on the membrane permeability inferred from exchange times measured with time‐dependent diffusion MRI because they increase the effective membrane surface area available for exchange relative to the enclosed volume. Because the inferred permeability scales approximately as $\xi \propto \frac{k_{\mathrm{ex}}}{SV}$, an increase in surface‐to‐volume ratio lowers the permeability required to explain a given measured exchange rate. Consequently, if dendrites are modeled as smooth shafts and the membrane area contributed by spines is ignored, the estimated permeability will be biased upward (i.e., overestimated). This effect may be particularly important in highly spiny dendrites, where a significant fraction of the membrane resides in spine heads and necks rather than in the shaft itself. More generally, the correction introduced by spines is expected to be predominantly geometric, although it should likely be regarded as an upper bound because the narrow spine neck can restrict communication between the spine head and the shaft, so that not all the added membrane may contribute equally over the diffusion time scale of the experiment.

As an illustrative example, consider a dendritic shaft of radius 0.5μm. For a L=1μm long shaft segment, the surface area is $S_{\mathrm{sh}}=2\mathrm{\pi rL}=3.14\;\mu m^{2}$ and the volume is $V_{\mathrm{sh}}=\pi r^{2}L=0.785\;\mu m^{3}$, yielding $SV_{\mathrm{sh}}=4.0\;\mu m^{-1}$. Assuming spines with head radius 0.4μm, neck radius 0.125μm, neck length 1.2μm, and a realistic spine density ρ between 0.6 and 2.5 spines per μm, each spine contributes approximately $2.86\;\mu m^{2}$ of exposed membrane area and $0.33\;\mu m^{3}$ of volume. Under these assumptions, the effective surface‐to‐volume ratio increases $4.95-6.41\;\mu m^{‐1}$, corresponding to a 24%‐60% increase relative to the smooth shaft alone. Because the inferred membrane permeability scales as $\xi \propto \frac{k_{\mathrm{ex}}}{SV}$, inclusion of spines reduces the estimated permeability to 0.81−0.62  of the shaft‐only value. For example, if $k_{\mathrm{ex}}=0.02\;ms^{-1}$, the inferred permeability decreases from $5\times 10^{-3}\frac{\mathrm{\mu m}}{\mathrm{ms}}$ for the smooth‐shaft model down to $\sim 3\times 10^{-3}\frac{\mathrm{\mu m}}{\mathrm{ms}}$ when spine geometry is included.

### 5.4 Perpendicular Signal Component in Dendritic Spines

In general, the perpendicular signal component of diffusing water molecules in the GM is considered negligible due to small radii of neurites [13, 14, 16, 39, 112, 113, 114] and motional averaging of the diffusion process in the clinical measurements [14, 16, 110, 111]. Consequently, neurites in GM are often assumed to exhibit nondecaying perpendicular diffusion signals and are modeled as randomly oriented sticks. However, studies employing ultra‐high diffusion‐weighting have not provided strong evidence supporting the validity of the stick model in GM [113, 114, 135]. On the contrary, the presence of dendritic spines may violate this assumption by increasing the effective fiber radius at higher spine densities [131], thereby introducing additional signal bias and q−t dependence in the direction perpendicular to the main axis of the shaft. In this study, while our primary investigation focuses on the diffusion and exchange dynamics of dendritic spines, we also wish to highlight an important observation from our simulations: the nonnegligible signal decay of the perpendicular signal component. We believe this finding is of interest to the diffusion community. Examining the perpendicular signal component of the Monte Carlo simulations, we found that there is considerable signal attenuation up to 40% for typical spine densities ($0.5‐3.0\;\mu m^{‐1}$) at short and moderate diffusion times. Figure S6 shows the perpendicular signal attenuations ($S_{⊥}$) for several spine densities from the Monte Carlo simulations with narrow (A) and wide (B) pulses. The signal decay in the motional averaging regime [110, 111] varies between 10% and 20% for typical spine densities (see Figure S6B), indicating that $S_{⊥}$ has a nonnegligible impact in the diffusion analysis. Moreover, Figure 10 shows the model fitting of $S_{⊥}$ using the Neuman approximation [115] in the coarse‐graining regime, demonstrating that estimated effective fiber radii are sensitive to total spine volume fraction (or density) (Figure S7). The larger effective dendritic branch size due to the presence of spines agrees with previous work studying the spines and leaflets [131]. Palombo et al. reported larger branch sizes estimated due to the total spine length and density. Therefore, the potential signal attenuation in the direction perpendicular to the neurites might be significant in dMRI measurements due to the dendritic spines in the GM. To address this potential bias in the context of exchange estimation, experimental designs that vary the exchange encoding while keeping the restriction encoding constant would be advised [31].

### 5.5 Limitations

We acknowledge a few limitations of the current study. Our digital models do not include various spine populations in addition to mushroom‐like, which are instead typically present in the real branches, such as immature filopodia or spike‐like spines. In addition, our simulations were performed in spiny dendritic branches with varying spine density, but do not accurately simulate the macroscopic voxel. Hence, our findings resulted from oversimplified simulations. For instance, while previous studies [7, 13] have reported negligible or indistinguishable effects of cell bodies on exchange dynamics between the cell body and neurites, our analysis does not account for the restriction effects of cell bodies on the diffusion process. More realistic diffusion simulations might be achieved by employing numerical gray matter phantoms [136] after the inclusion of dendritic spines.

Our diffusion simulations were designed with simple diffusion encodings [41] and studied short and wide pulse acquisitions. Although our results align with the literature regarding the underestimation observed in exchange rates with wide pulses [14, 42], the degeneracy in estimating diffusion metrics of restriction and exchange remained unresolved in our SDE‐based simulations [13, 14, 16, 44, 137]. More advanced acquisition techniques address this issue by incorporating additional experimental dimensions [16, 30, 31, 33, 54], which may help resolve the degeneracy between total spine volume fraction and membrane permeability observed in estimated exchange rates in our analysis, and it is the focus of another study [55] which complements the present one.

Finally, the proposed extended Kärger model of three compartments neglects the diffusion signal decay in the dendritic spines and considers it as dot compartment for simplification. This assumption in our proposed model might overestimate the restriction effect on the molecular diffusion process. Further corroborations are required to assess the measurable diffusion in dendritic spines and its potential impact on the interplay between restriction and exchange.

## 6 Conclusion

Our study demonstrates that the time‐dependent SDE signal arising from diffusion within impermeable spiny dendrites is indistinguishable from that resulting from permeative exchange. We show that a modified two‐compartment Kärger model accurately captures the time‐dependent SDE signal along the dendritic shaft in spiny dendritic branches but yields biased estimates of exchange rates. We proposed an extended three‐compartment Kärger model, which incorporates the contribution of dendritic spines, to simulate exchange between dendritic shaft, dendritic spines and extracellular space. Our analysis using the extended Kärger model revealed that diffusion‐mediated exchange within spiny dendrites can introduce bias in NEXI/SMEX estimates of exchange rate. Additionally, we proposed an adaptation of the extended Kärger model to the coarse‐graining regime, simplifying the biophysical modelling. Therefore, the presence of spiny dendrites and unaccounted diffusion‐mediated exchange can bias NEXI/SMEX estimates of permeative exchange in gray matter, depending on the total spine volume fraction. Although useful to study the interweaved impact of both diffusion‐mediated and permeative exchange, the proposed three‐compartment Kärger model and its coarse‐grained version cannot disentangle the respective contributions of membrane permeability and total spine volume fraction. Furthermore, our results show that neglecting dendritic spines can systematically overestimate dendritic membrane permeability, emphasizing the need to incorporate realistic spine geometry in the interpretation of exchange‐sensitive diffusion MRI measurements.

In conclusion, we highlight the need for careful interpretation of diffusion MRI‐based exchange estimates, particularly when attributing them solely to membrane permeability, and highlight the unmet need for better methods and modelling approaches to disentangle permeative and diffusion‐mediated exchange mechanisms.

## Author Contributions

**Kadir Şimşek:** conceptualization, formal analysis, methodology, investigation, writing – original draft. **Arthur Chakwizira:** writing – review and editing. **Markus Nilsson:** writing – review and editing. **Marco Palombo:** conceptualization, methodology, funding acquisition, writing – original draft, supervision.

## Funding

This study was supported by the UKRI Future Leaders Fellowship (MR/T020296/2), the Swedish Research Council (2024‐04968), and Hjärnfonden (FO2024‐0335‐HK‐73).

## Conflicts of Interest

The authors declare no conflicts of interest.

## Supporting information


**Data S1:** Supporting Information.

**Figure S1:** Normalized particle concentration curves illustrating shaft‐to‐spine and spine‐to‐shaft particle dynamics. Shaft‐to‐spine exchange is substantially faster than spine‐to‐shaft exchange. The 50% concentration level (red horizontal line) is used to characterize the diffusive exit rates for each compartment. Shaft‐to‐spine dynamics are sensitive to spine volume fraction (or density), consistent with theoretical expectations, whereas spine‐to‐shaft dynamics are largely independent of spine density, being primarily determined by spine morphology.

**Figure S2:** (A) Illustration of the equivalent 1D permeable‐membrane model. Membranes were positioned at locations corresponding to randomly distributed spine attachment sites along the dendritic branch. The histogram shows the resulting inter‐membrane spacing distribution for a spine density of $\left(\rho =0.6\;\mu m^{-1}\right),$ with a mean spacing of 1.6 μm. (B) Simulated diffusion‐weighted signals obtained from the 1D permeable‐membrane model using the narrow‐pulse encoding scheme.

**Figure S3:** The diffusion signals in the parallel direction obtained from undulated and beaded synthetic substrates with spine density $\rho =1\;\mu m^{-1}$ are shown in the figure for narrow (A) and wide (B) pulse schemes. The signals from undulated substrates show alignment with the modified Kärger model for diffusion times $t_{d}\leq 54\;\mathrm{ms}$, for $t_{d}>54\;\mathrm{ms}$, the restriction effect starts dominating the diffusion signal. On the contrary, the diffusion signals obtained from any beaded substrates exhibit the dominance of the restriction over exchange effect at all diffusion times.

**Figure S4:** Powder‐averaged diffusion‐weighted signals were generated using 128 gradient directions and *b* values ranging from 3 to $20\;\frac{\mathrm{ms}}{\mu m^{2}}$ for both narrow and wide pulse schemes. Color coding indicates diffusion time, spanning up to 34 ms for the narrow‐pulse scheme and up to 50 ms for the wide‐pulse scheme. Exchange‐driven signal behavior is evident for $t_{d}\leq 24\;\mathrm{ms}$, whereas the wide‐pulse scheme is dominated by restriction effects owing to motional averaging during the gradient pulse.

**Figure S5:** The axial diffusivity ($D_{∥}$) was measured from the particle displacement (Einstein, 1905; Jensen et al., 2005; Kiselev, 2010) for the spiny dendritic substrates and parametrized for plausible total spine volume fractions. A clear linear relation between total spine volume fraction and $D_{\text{in}}$ is shown in the figure. Later, we informed $D_{\text{in}}$ in NEXI/SMEX model fitting to “correct” the bias induced from dendritic spines.

**Figure S6:** The perpendicular signal component ($S_{⊥}$) of the simulated diffusion signals for several spine densities are shown in the figure for narrow (A) and wide (B) gradient pulses. For typical spine densities, the signal attenuation $S_{⊥}$ is not negligible and can be up to 40%, especially at short and moderate diffusion times. Solid lines represent fits to Neuman's approximation for diffusion in impermeable cylinders (Neuman, 1974; Veraart et al., 2020). The model accurately describes the simulated signals except in regimes where the long‐pulse and/or Gaussian phase approximations underlying the theory are not strictly fulfilled.

**Figure S7:** Radial apparent diffusion coefficient, $\mathrm{AD}C_{⊥}$, of spiny‐dendrite simulations as a function of diffusion time for increasing spine densities ρ ($0.0‐3.0\;\mu m^{‐1}$; color‐coded). **Left:** $\mathrm{AD}C_{⊥}$ plotted against diffusion time $t_{d}$, showing a monotonic decrease with $t_{d}$ that grows in magnitude with spine density. **Right:** $\mathrm{AD}C_{⊥}$ plotted against ${t_{d}^{-}}^{1}$ to examine the radial diffusion‐length scaling $\frac{R^{2}}{4t_{d}}$ at long diffusion times ($t_{d}\geq 24\;\mathrm{ms}$). The estimated $\mathrm{AD}C_{⊥}$ scales linearly with $t_{d}^{-1}$, yielding effective perpendicular compartment sizes $R_{\mathrm{eff}}$ that increase with spine density (0.23‐1.77 μm). This confirms that spines break the stick assumption, giving the dendrite a finite effective radius in the coarse‐grained regime.

## Data Availability

All the data and analysis codes underpinning the results presented here can be found upon publication in the Cardiff University data catalogue and on Github: https://github.com/kdrsimsek/dendriticSpines_exchange.
