# Supplementary material for: The Role of Dendritic Spines in Water Exchange Measurements With Diffusion MRI: Time‐Dependent Single Diffusion Encoding MRI
Source: NMR Biomed. 2026 Sep 11;39(10):e70382. doi: 10.1002/nbm.70382 (PMC13569403; doi:10.1002/nbm.70382)
Supplement: Supplementary file 1 [file NBM-39-e70382-s004.docx]

# **Supplementary Figures**

**Figure S1:** Normalized particle concentration curves illustrating shaft-to-spine and spine-to-shaft particle dynamics. Shaft-to-spine exchange is substantially faster than spine-to-shaft exchange. The 50% concentration level (red horizontal line) is used to characterize the diffusive exit rates for each compartment. Shaft-to-spine dynamics are sensitive to spine volume fraction (or density), consistent with theoretical expectations, whereas spine-to-shaft dynamics are largely independent of spine density, being primarily determined by spine morphology.

**Figure S2**: (A) Illustration of the equivalent 1D permeable-membrane model. Membranes were positioned at locations corresponding to randomly distributed spine attachment sites along the dendritic branch. The histogram shows the resulting inter-membrane spacing distribution for a spine density of $(\rho=0.6 \mu m^{-1}),$ with a mean spacing of $1.6 \mu m$. (B) Simulated diffusion-weighted signals obtained from the 1D permeable-membrane model using the narrow-pulse encoding scheme.

**Figure S3:** The diffusion signals in the parallel direction obtained from undulated and beaded synthetic substrates with spine density $\rho=1 \mu m^{-1}$ are shown in the figure for narrow (A) and wide (B) pulse schemes. The signals from undulated substrates show alignment with the modified Kärger model for diffusion times $t_{d}<150 ms$, for $t_{d}>150 ms$, the restriction effect starts dominating the diffusion signal. On the contrary, the diffusion signals obtained from any beaded substrates exhibit the dominance of the restriction over exchange effect at all diffusion times.

**Figure S4**: Powder-averaged diffusion-weighted signals were generated using 128 gradient directions and b-values ranging from $3$ to $20\frac{\mu m^{2}}{\mathrm{ms}}$ for both narrow and wide pulse schemes. Color coding indicates diffusion time, spanning up to 34 ms for the narrow-pulse scheme and up to $50 \mathrm{ms}$ for the wide-pulse scheme. Exchange-driven signal behavior is evident for $t_{d}\leq24 \mathrm{ms}$, whereas the wide-pulse scheme is dominated by restriction effects owing to motional averaging during the gradient pulse.

**Figure S5:** The axial diffusivity ($D_{\parallel}$) were measured from the particle displacement (Einstein, 1905; Jensen et al., 2005; Kiselev, 2010) for the spiny dendritic substrates and parametrized for plausible total spine volume fractions. A clear linear relation between total spine volume fraction and $D_{in}$ is shown in the figure. Later, we informed $D_{in}$ in NEXI/SMEX model fitting to “correct” the bias induced from dendritic spines.

**Figure S6**: The perpendicular signal component ($S_{\perp}$) of the simulated diffusion signals for several spine densities are shown in the figure for narrow (A) and wide (B) gradient pulses. For typical spine densities, the signal attenuation $S_{\perp}$is not negligible and can be up to 30%, especially at short and moderate diffusion times. Solid lines represent fits to Neuman's approximation for diffusion in impermeable cylinders (Neuman, 1974; Veraart et al., 2020). The model accurately describes the simulated signals except in regimes where the long-pulse and/or Gaussian phase approximations underlying the theory are not strictly fulfilled.

**Figure S7**: Radial apparent diffusion coefficient, $ADC_{\perp}$, of spiny-dendrite simulations as a function of diffusion time for increasing spine densities $\rho$ ($0.0-3.0 \mu m⁻¹;$ color-coded). **Left:** $ADC_{\perp}$ plotted against diffusion time $t_{d}$, showing a monotonic decrease with $t_{d}$ that grows in magnitude with spine density. **Right:** $ADC_{\perp}$ plotted against ${t_{d}^{-}}^{1}$ to examine the radial diffusion-length scaling $\frac{R^{2}}{4t_{d}}$at long diffusion times ($t_{d} \geq24 ms$). The estimated $ADC_{\perp}$ scales linearly with $t_{d}^{-1}$, yielding effective perpendicular compartment sizes $R_{eff}$ that increase with spine density ($0.23-1.77 \mu m$). This confirms that spines break the stick assumption, giving the dendrite a finite effective radius in the coarse-grained regime.

# **References**

Einstein, A. (1905). Über die von der molekularkinetischen Theorie der Wärme geforderte Bewegung von in ruhenden Flüssigkeiten suspendierten Teilchen. *Annalen Der Physik*, *322*(8), 549–560. https://doi.org/10.1002/ANDP.19053220806

Jensen, J. H., Helpern, J. A., Ramani, A., Lu, H., & Kaczynski, K. (2005). Diffusional kurtosis imaging: The quantification of non-Gaussian water diffusion by means of magnetic resonance imaging. *Magnetic Resonance in Medicine*, *53*(6), 1432–1440. https://doi.org/10.1002/mrm.20508

Kiselev, V. G. (2010). The Cumulant Expansion: An Overarching Mathematical Framework For Understanding Diffusion NMR. *Diffusion MRI*, 152–168. https://doi.org/10.1093/MED/9780195369779.003.0010

Neuman, C. H. (1974). Spin echo of spins diffusing in a bounded medium. *The Journal of Chemical Physics*, *60*(11), 4508–4511. https://doi.org/10.1063/1.1680931

Novikov, D. S., Jensen, J. H., Helpern, J. A., & Fieremans, E. (2014). Revealing mesoscopic structural universality with diffusion. *Proceedings of the National Academy of Sciences of the United States of America*, *111*(14), 5088–5093. https://doi.org/10.1073/PNAS.1316944111/ASSET/1368C237-0C8F-4A2E-B26E-B84C3DF6D319/ASSETS/GRAPHIC/PNAS.1316944111I93.GIF

Veraart, J., Nunes, D., Rudrapatna, U., Fieremans, E., Jones, D. K., Novikov, D. S., & Shemesh, N. (2020). Noninvasive quantification of axon radii using diffusion MRI. *ELife*, *9*. https://doi.org/10.7554/ELIFE.49855
